# OsSAE1 orchestrates the antagonistical regulation of gibberellin and abscisic acid signaling to control rice seed germination

**DOI:** 10.1111/jipb.70062

**Published:** 2025-10-30

**Authors:** Dinglin Xiao, Yuxiang Li, Bingkun Ge, Zihan Zhao, Zhiheng Suo, Juan Wang, Chunxia Yan, Zhi Qi, Rongfeng Huang, Ruidang Quan, Hua Qin

**Affiliations:** ^1^ Biotechnology Research Institute Chinese Academy of Agricultural Sciences Beijing 100081 China; ^2^ Key Laboratory of Forage and Endemic Crop Biology, Ministry of Education, School of Life Science Inner Mongolia University Hohhot 010021 China; ^3^ National Key Facility of Crop Gene Resources and Genetic Improvement Beijing 100081 China; ^4^ Shandong Provincial Key Laboratory of Plant Stress College of Life Sciences, Shandong Normal University Jinan 250014 China

**Keywords:** antagonism of ABA−GA signaling, AP2 transcription factor OsSAE1, elite haplotype, rice direct seeding, seed germination

## Abstract

The plant life cycle and the promise of crop yield start with successful seed germination, which requires an optimal balance between the phytohormones abscisic acid (ABA) and gibberellin (GA). Here, we report that the APETALA 2‐type transcription factor SALT AND ABA RESPONSE ERF 1 (OsSAE1) antagonistically modulates ABA and GA signaling to control seed germination in rice (*Oryza sativa* L.). We show that knocking out *OsSAE1* delays seed germination, concomitant with the accumulation of SLENDER RICE1 (OsSLR1), a GA signaling repressor DELLA protein; importantly, GA application rescued the seed germination defect of *ossae1* mutants. OsSAE1 directly activates transcription of the GA biosynthesis gene *OsKS1* and represses that of the GA metabolism gene *OsGA2ox3*, resulting in higher GA levels. Moreover, OsSLR1 physically interacts with ABA‐INSENSITIVE 5 (OsABI5), a key ABA signaling component, enhancing the transcriptional activation capacity of OsABI5 toward its target genes to regulate seed germination. The temporal expression pattern of *OsSAE1* supports its role in orchestrating GA and ABA signaling to modulate seed germination and seed dormancy. Different *OsSAE1* haplotypes differentially affected *OsSAE1* transcript levels and seed germination rates, illustrating the potential of the elite *OsSAE1* haplotype for genetic improvement of seed germination. Overall, our study reveals that OsSAE1 controls rice seed germination by regulating the balance between ABA and GA, providing a pivotal selection target for breeding rice cultivars suitable for direct seeding.

## Graphical abstract

The AP2 transcription factor SALT AND ABA RESPONSE ERF 1 (OsSAE1) acts as a key regulator in improving rice seed germination rate. OsSAE1 precisely controls the homeostasis of abscisic acid and gibberellin in seeds, and provides a target for breeding rice varieties that are suitable for direct seeding.

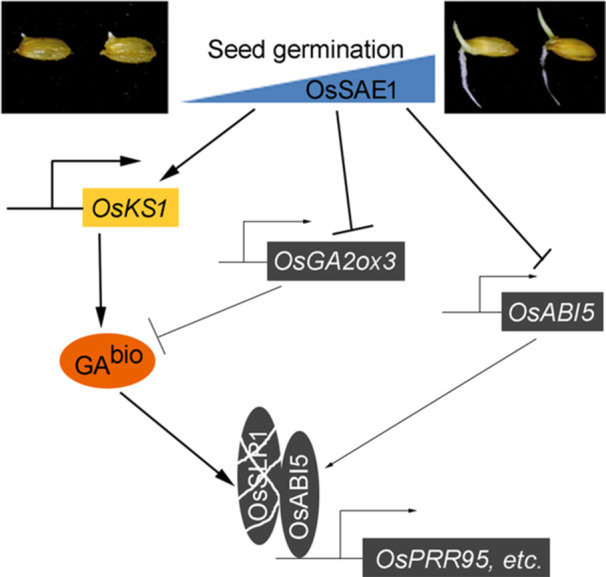

## INTRODUCTION

Seed germination initiates the plant life cycle and largely dictates plant development and crop production. In fact, rapid and uniform seed germination often confers clear growth advantages and greater resistance to abiotic stresses upon the resulting seedlings and plants, thus diminishing seedling mortality and raising yield (Rajjou et al., 2012; Mahender et al., 2015; He et al., 2019). Rice (*Oryza sativa* L.) is a major crop cultivated worldwide. Direct rice seeding offers lower financial and labor costs than traditional transplantation practices (Farooq et al., 2011; Liu et al., 2015). However, direct seeding requires high seed germination rates in the field to be effective (Mahender et al., 2015). Thus, identifying key genes associated with seed germination and dissecting their underlying mechanisms are prerequisites before any efforts to genetically improve seed germination can be undertaken through rice breeding.

Seed germination is regulated by environmental cues and intrinsic signals, in particular diverse phytohormones (Shu et al., 2016a; Yang et al., 2020; Sajeev et al., 2024; Xu et al., 2025). Abscisic acid (ABA) and gibberellin acid (GA) are the two primary phytohormones that antagonistically regulate seed germination (Shu et al., 2013, 2016b). Indeed, plants with mutation or overexpression (OE) of genes involved in ABA and GA biosynthesis or signaling often suffer from abnormal seed germination rates (Ma et al., 2009; Wang et al., 2020; Zhang et al., 2020; Xing et al., 2023). Extensive studies have shown that ABA biosynthesis and signaling are repressed during the seed imbibition stage, whereas GA biosynthesis and signaling are activated (Finkelstein et al., 2008; Shu et al., 2016a; Penfield, 2017; Sajeev et al., 2024), suggesting that precise ABA and GA homeostasis in seeds is a critical determinant of seed germination.

There is complex crosstalk between ABA and GA signaling pathways during seed germination (Shu et al., 2013, 2016a; Xian et al., 2024), and several factors have been identified that might be involved in the antagonistic effects of these two phytohormones. For example, Arabidopsis MYELOBLASTOSIS 96 (AtMYB96) transcription factor negatively regulates seed dormancy by repressing the expression of genes related to ABA biosynthesis and promoting that of genes associated with GA biosynthesis (Lee et al., 2015). In rice, *PYRUVATE KINASE 5* (*OsPK5*) improves seed germination by affecting glycolytic metabolism and the ABA/GA balance (Yang et al., 2022). The phosphatidylethanolamine‐binding protein MOTHER OF FT AND TFL1 (OsMFT1) prevents seed germination under salinity stress by modulating ABA signaling and GA biosynthesis (Lu et al., 2023). These studies demonstrate the importance of ABA and GA biogenesis and signaling in the control of seed dormancy and seed germination.

APETALA 2 domain‐containing proteins/ethylene response factors (AP2/ERFs) define a large family of primarily plant‐specific transcription factors (Nakano et al., 2006), serving as important regulators of various stages during plant development and in response to multiple biotic and abiotic stresses (Liu et al., 2022; Li et al., 2022b; Wu et al., 2022b; Lim et al., 2024). Substantial evidence indicates that AP2/ERF transcription factors contribute to the antagonism between ABA and GA (Shu et al., 2018; Fu et al., 2021). For example, ABA‐INSENSITIVE 4 (AtABI4), an AP2 transcription factor in the ABA signaling pathway, represses seed germination by regulating the biogenesis of ABA and GA (Shu et al., 2013). In addition, RGA‐LIKE 2 (AtRGL2), a repressor of GA signaling, interacts with and stabilizes AtABI4 to enhance the transcriptional activation ability of AtABI4 toward its target genes to regulate seed germination (Xian et al., 2024). Similarly, the maize AP2/ERF member ZmEREB20 controls seed germination under salinity stress by regulating the expression of ABA‐related and GA‐related genes (Fu et al., 2021). The rice AP2‐like transcription factor OsAP2‐39 directly controls the expression of *9‐CIS‐EPOXYCAROTENOID DIOXYGENASE 1* (*OsNCED1*), a key ABA biosynthetic gene, as well as that of *ELONGATION OF UPPER MOST INTERNODE 1* (*EUI1*), encoding a GA deactivation enzyme to regulate plant growth and seed production (Yaish et al., 2010).

We previously showed that the AP2/ERF protein SALT AND ABA RESPONSE ERF1 (OsSAE1) positively regulates seed germination by repressing *OsABI5* transcription (Li et al., 2022b). Here, we explored whether AP2/ERF transcription factors, such as OsSAE1, modulate rice seed germination by mediating the GA–ABA antagonism. We determined that OsSAE1 antagonistically monitors the ABA and GA signaling pathways to regulate rice seed germination, providing a key selection target for breeding rice cultivars suitable for direct seeding.

## RESULTS

### Gibberellin is required for OsSAE1‐regulated seed germination

**Figure 1:**
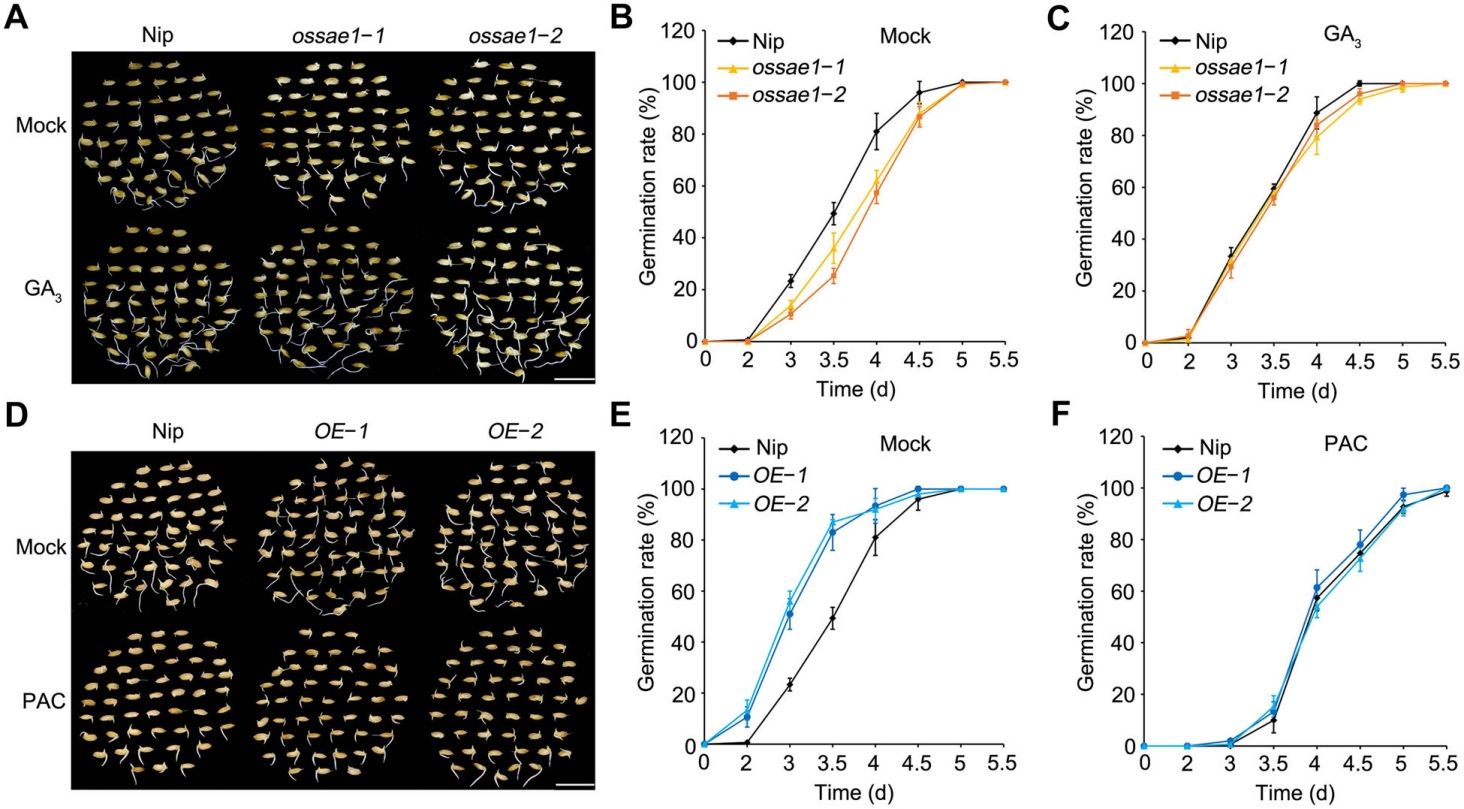
Exogenous gibberellin (GA_3_) or paclobutrazol treatment recovers the phenotype of seed germination in *OsSAE1* OE and knockout lines **(A)** Representative images of seed germination among Nipponbare (Nip) and *ossae1* mutants with or without 5 μmol/L GA_3_ treatment for 3.5 d. Scale bar, 1 cm. **(B**, **C)** Dynamic of germination rate among Nip and *ossae1* mutants with **(C)** or without 5 μmol/L GA_3_ **(B)** treatment. The data are shown as mean ± *SD*, *n* = 3 biological replicates. **(D)** Representative images of seed germination among Nip and *OsSAE1*‐*OE* lines with or without 2 μmol/L paclobutrazol (PAC) treatment for 3.5 d. Scale bar, 1 cm. **(E**, **F)** Dynamic of germination rate among Nip and *OsSAE1*‐*OE* lines with **(F)** or without 2 μmol/L PAC **(E)** treatment. The data are shown as mean ± *SD*, *n* = 3 biological replicates.

Gibberellin regulates seed germination, and disrupting GA biosynthesis or signaling leads to delayed seed germination (Claeys et al., 2014; Sánchez−Montesino et al., 2019; Xing et al., 2023). In previous work, we showed that OsSAE1 positively regulates seed germination (Li et al., 2022b). To examine whether OsSAE1‐mediated regulation of seed germination involves the GA pathway, we tested seed germination in the presence of GA_3_ or the GA biosynthesis inhibitor paclobutrazol (PAC). We determined that the germination rate of *OsSAE1‐OE* seeds under normal conditions was significantly higher than that of seeds from the wild‐type Nipponbare (Nip), whereas *ossae1* seeds exhibited a lower germination rate than that of Nip (Figure S1A, B), consistent with our previous report (Li et al., 2022b). Adding 5 μmol/L GA_3_ to the medium completely rescued the defect in seed germination of the *ossae1* mutants (Figure 1A−C). Similarly, the faster seed germination rate seen in *OsSAE1‐OE* seeds was largely counteracted by treatment with 2 μmol/L PAC (Figure 1D−F). These results suggest that GA participates in OsSAE1‐promoted seed germination.

Since α‐amylase and starch mobilization are important modulating factors in seed germination, and as GA induces the expression of α‐amylase genes for starch degradation in the seeds of cereal crops (Gómez‐Cadenas et al., 2001; Washio, 2003; Asatsuma et al., 2005; Zhang et al., 2020; Xiong et al., 2022), we examined α‐amylase activity in Nip, *ossae1*, and *OsSAE1*‐*OE* seeds. We first assessed α‐amylase activity qualitatively via the starch plate assay. Specifically, the starch present in the agar medium is degraded by α‐amylases biosynthesized by dissected half seeds lacking the embryo, leading to a colorless halo around the seed half. Halo size is positively associated with α‐amylase activity in seeds. The starch plate assays revealed a larger starch‐free clear zone around *OsSAE1*‐*OE* seeds than around Nip seeds, and even smaller clear halos around *ossae1* seeds (Figure S1C, D). Following quantification of α‐amylase activity in germinating seeds with a starch assay kit, we measured higher α‐amylase activity in *OsSAE1*‐*OE* seeds and lower activity in *ossae1* seeds compared with that in Nip seeds (Figure S1E). In agreement with these results, α‐amylase genes were expressed to higher levels in *OsSAE1*‐*OE* seeds and to lower levels in *ossae1* seeds than in Nip seeds (Figure S1F, G). These results indicate that OsSAE1 affects the transcript levels of α‐amylase genes to modulate seed germination through GA signaling.

### OsSAE1 directly regulates *OsKS1* and *OsGA2ox3* expression involved in seed germination

**Figure 2:**
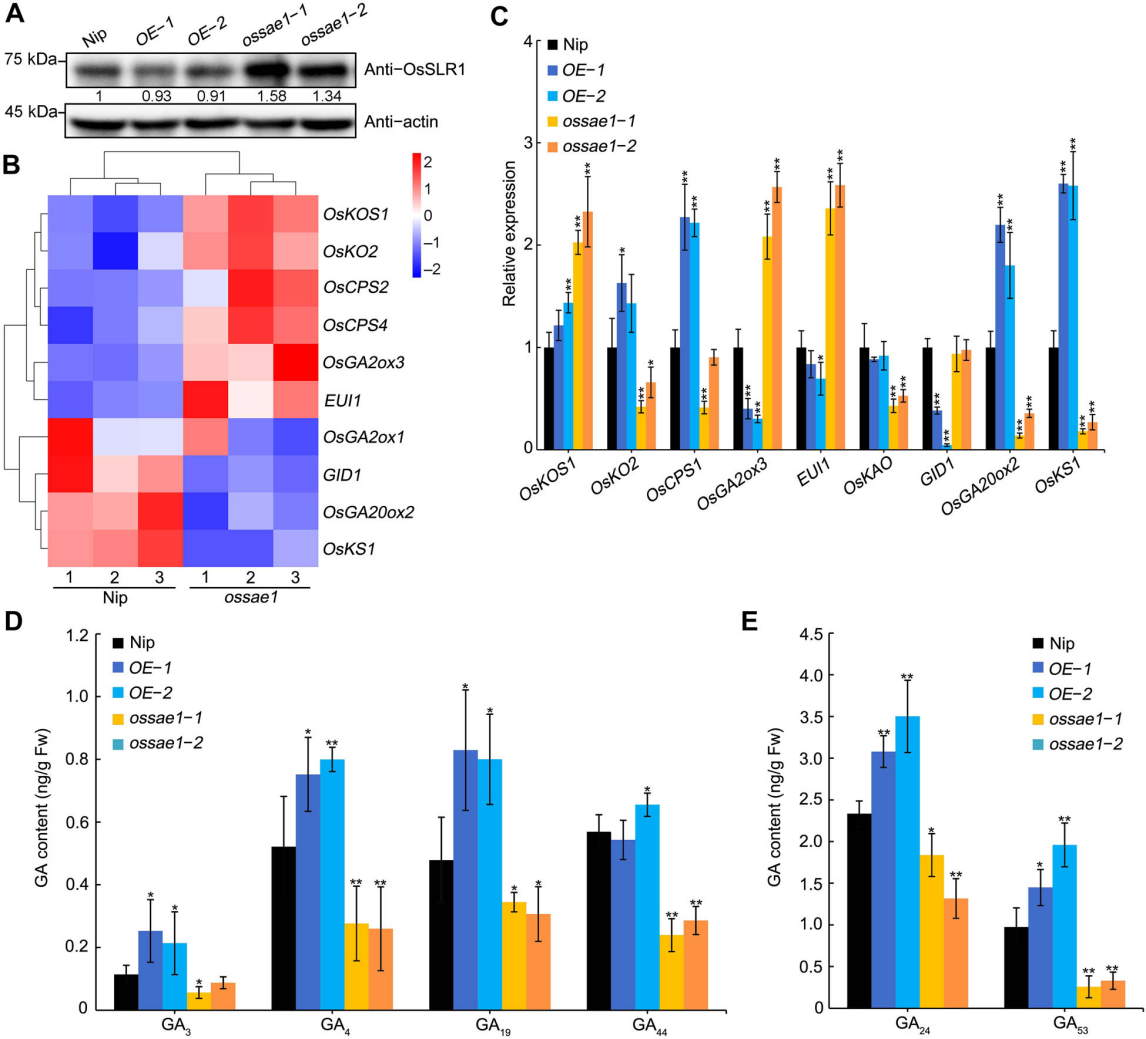
OsSAE1 regulates the expression of GA‐related genes to modulate GA accumulation **(A)** SLENDER RICE1 (OsSLR1) protein levels in Nipponbare (Nip), *ossae1* mutants and *OsSAE1*‐*OE* lines. Anti‐OsSLR1 antibody was used to detect protein levels of OsSLR1. Actin was used as a loading control. **(B)** Heat map of microarray expression profiles for GA biosynthesis, deactivation and signaling‐related genes. **(C)** Reverse transcription quantitative polymerase chain reaction (RT‐qPCR) analysis of GA biosynthesis, deactivation and signaling‐related genes in Nip, *ossae1* mutants and *OsSAE1*‐*OE* lines. Seeds germinated for 2 d after imbibition were used for RNA extraction. *Actin1* was used as an internal control. The relative expression levels are represented by fold change relative to the expression levels of Nip. **(D**, **E)** Content of various GAs in the seeds of Nip, *ossae1* mutants and *OsSAE1*‐*OE* lines. GAs were extracted from embryo‐less seeds that germinated 2 d after imbibition. For **(C–E)**, data are presented as mean ± *SD*, *n* = 3 biological replicates. Asterisks indicate significant differences compared with Nip at *P < 0.05 and **P < 0.01 (Student's *t*‐test).

*ossae1* seeds showed lower α‐amylase activity than Nip seeds, and application of GA_3_ rescued the defect in seed germination, suggesting a link between OsSAE1 and GA accumulation. To test this hypothesis, we examined the abundance of OsSLR1 in seeds, as OsSLR1 degradation is promoted by GA (Ueguchi‐Tanaka et al., 2008). Although the expression levels of *OsSAE1* were six‐ to seven‐fold higher in the *OsSAE1*‐*OE* lines, *OsSLR1* expression levels were comparable among the two *OsSAE1*‐*OE* lines and Nip (Figure S2). An immunoblot analysis detected a lower abundance for OsSLR1 in *OsSAE1*‐*OE* seeds, while OsSLR1 accumulated to higher levels in *ossae1* mutants, compared with its levels in Nip seeds (Figure 2A). This result suggests that the levels of bioactive GAs are higher in *OsSAE1*‐*OE* seeds but lower in *ossae1* seeds than in Nip seeds and that OsSLR1 may be subjected to GA‐induced degradation.

To elucidate the molecular network underlying OsSAE1‐regulated GA accumulation, we examined the expression levels of genes associated with GA biosynthesis, deactivation, or signaling in our previous transcriptome deep sequencing (RNA‐seq) dataset (PRJNA793282) (Li et al., 2022b). Indeed, several genes related to GA biosynthesis (*OsGA20ox2*, *OsKS1*) were downregulated in the *ossae1* mutant, whereas genes involved in GA deactivation (*OsGA2ox3*, *EUI1*) were upregulated (Figure 2B). Reverse transcription quantitative polymerase chain reaction (RT‐qPCR) analysis showed that OsSAE1 positively regulates the expression of *OsGA20ox2* and *OsKS1*, but negatively regulates that of *OsGA2ox3* in germinating seeds (Figure 2C), suggesting that OsSAE1 may possess both transcriptional activation and repression activities. To test this idea, we performed a transactivation activity assay in yeast cells and found that OsSAE1 exhibited transcriptional activation activity in yeast cells (Figure S3A). To examine the repression activity potential of OsSAE1, we cloned the full‐length coding region of OsSAE1 in‐frame and downstream of the sequence for the strong transcriptional activator VP16, itself placed downstream of the GAL4 DNA‐binding domain, to generate GAL4DB‐VP16‐OsSAE1. According to our results, GAL4DB‐VP16‐OsSAE1 exhibited much lower relative luciferase (LUC) activity compared to the control GAL4DB‐VP16 (Figure S3B), indicating that OsSAE1 also possesses transcriptional repression activities. We then determined the GA contents in the seeds of Nip, *OsSAE1‐OE*, and *ossae1*. We detected higher levels of GA precursors (GA_19_, GA_24_, GA_44_, and GA_53_) and bioactive GAs (GA_3_ and GA_4_) in *OsSAE1‐OE* lines and lower levels in *ossae1* mutants than in Nip (Figure 2D, E). These results collectively suggest that OsSAE1 modulates the content of bioactive GAs, possibly by regulating the expression of GA biosynthesis and deactivation genes, raising the possibility that *OsGA20ox2*, *OsKS1*, and *OsGA2ox3* are potential target genes of OsSAE1.

**Figure 3:**
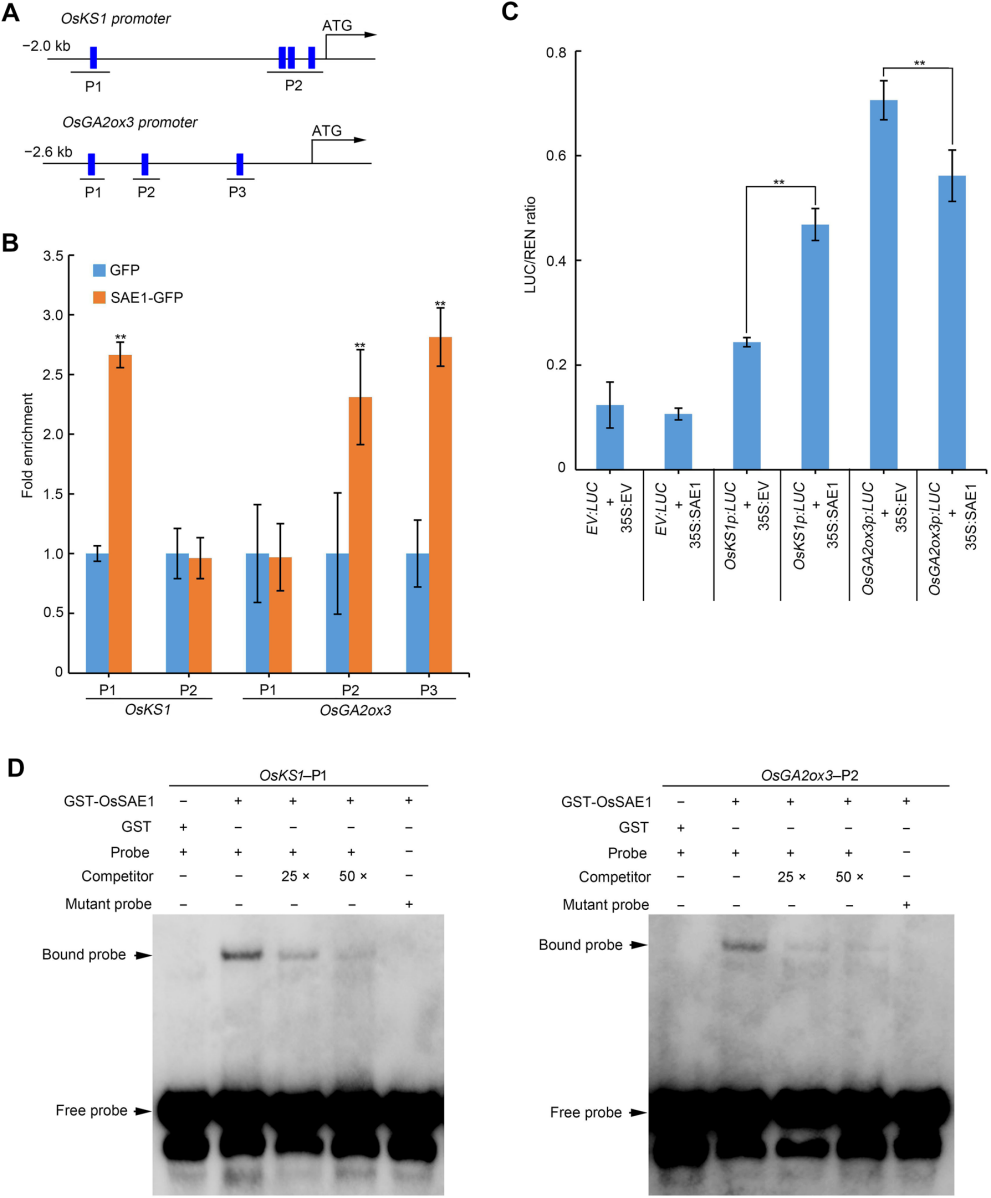
OsSAE1 directly binds to the promoter of *OsKS1* and *OsGA2ox3* to regulate their expression **(A)** Schematic diagram of GCC‐box in the *OsKS1* and *OsGA2ox3* promoter. P1, P2 and P3 are promoter fragments used in cleavage under targets and tagmentation quantitative polymerase chain reaction (CUT&Tag–qPCR) experiments. The blue boxes indicate GCC‐box and the black lines indicate the promoter sequence. **(B)** Normalized qPCR results showing the relative enrichment of fragments from indicated promoter regions of genes in the OsSAE1 assay group (OsSAE1‐GFP (green fluorescent protein)) compared with the control group (GFP). Data were presented as mean ± *SD*, *n* = 3 biological replicates. Asterisks indicate significant differences compared with GFP at **P < 0.01 (Student's *t*‐test). **(C)** Dual‐luciferase (LUC) assay in rice protoplasts using constructs constitutively expressing *OsSAE1* and/or the LUC reporter gene under control of the *OsKS1* or *OsGA2ox3* promoter. Data are presented as mean ± *SD*, *n* = 3 biological replicates. Asterisks indicate significant differences between the two compared samples using a Student's *t*‐test at **P < 0.01. **(D)** Electrophoretic mobility shift assay (EMSA) using normal (GCCGCC) and mutated (AAAAAA) *OsKS1* and *OsGA2ox3* promoter GCC‐box probes with glutathione S‐transferase (GST)‐tagged OsSAE1 (GST‐OsSAE1). GST‐tag was used in place of GST‐OsSAE1 for no‐protein controls. Protein was incubated with biotin‐labeled DNA fragments (Probe), tested for competition by adding an excess of unlabeled probe (Competitor), and for specificity with labeled mutant probe. Three biological replicates were performed, with similar results.

OsSAE1 can bind to the GCC‐box, a short *cis*‐acting element containing a core GCCGCC sequence motif (Li et al., 2022b). We analyzed the promoter sequences of *OsGA20ox2*, *OsKS1*, and *OsGA2ox3* and identified four GCC‐boxes in the *OsKS1* promoter and three GCC‐boxes in the *OsGA2ox3* promoter, but found no GCC‐box in the *OsGA20ox2* promoter region (Figure 3A). Hence, we performed a transient and simplified cleavage under targets and tagmentation (tsCUT&Tag) assay using protoplasts transfected with a *OsSAE1‐GFP* construct encoding a fusion of OsSAE1 and green fluorescent protein (GFP). We observed a significant enrichment of OsSAE1‐GFP at the P1 fragment of the *OsKS1* promoter and P2 and P3 fragments of the *OsGA2ox3* promoter (Figure 3B). We then carried out a transient *LUC* expression assay. The *LUC* reporter gene was driven by either the *OsKS1* or *OsGA2ox3* promoter and co‐transformed with *35S:OsSAE1* into rice protoplasts. In the presence of *35S:OsSAE1*, relative LUC activity derived from the *OsKS1* promoter was significantly induced compared with the LUC activity obtained with the empty effector vector, whereas the LUC activity derived from the *OsGA2ox3* promoter was significantly lower than that with the empty effector vector (Figure 3C). Thus, the presence of OsSAE1 promoted *OsKS1* transcription but suppressed that of *OsGA2ox3*. We asked whether OsSAE1 directly binds to the *OsKS1* and/or *OsGA2ox3* promoters by performing an electrophoretic mobility shift assay (EMSA) using recombinant purified glutathione S‐transferase (GST)‐OsSAE1. Indeed, the recombinant protein was able to bind directly to DNA probes containing the GCC‐box in the P1 fragment of the *OsKS1* promoter and to the P2 fragment of the *OsGA2ox3* promoter (Figure 3D). The binding was specific, as demonstrated by competition using unlabeled (competitor) and mutant probes (Figure 3D). These results indicate that OsSAE1 directly binds to the *OsKS1* and *OsGA2ox3* promoters to regulate their transcription.

**Figure 4:**
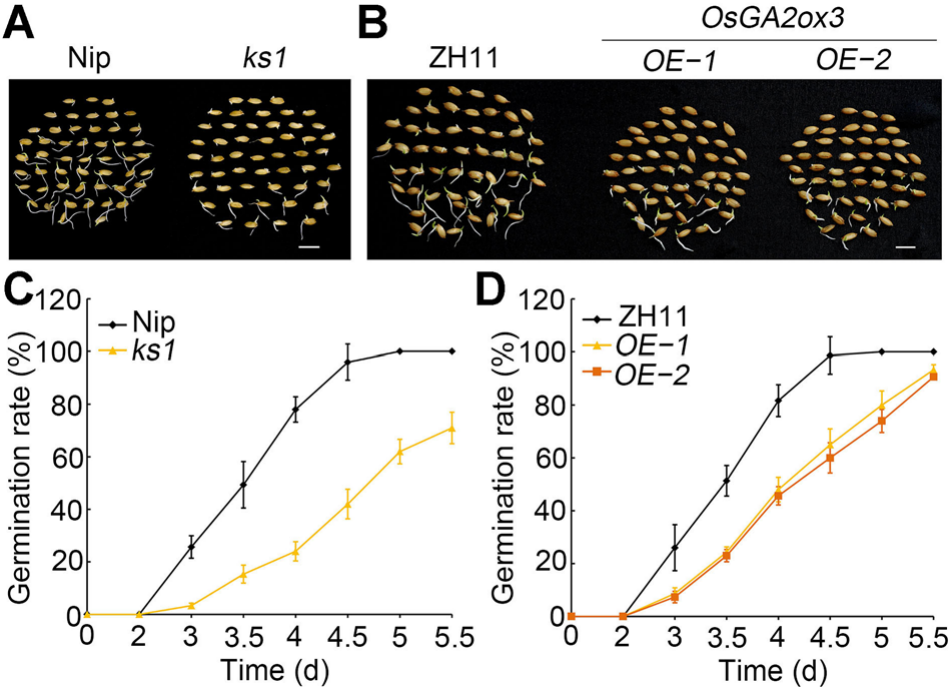
**Knockout** ***OsKS1*** **or overexpressing** ***OsGA2ox3*** **displayed delayed seed germination** **(A)** Representative images of seed germination performance of Nipponbare (Nip) and *ks1* mutant at 3.5 d. Scale bar, 1 cm. **(B)** Representative images of seed germination performance of Zhonghua11 (ZH11) and *OsGA2ox3*‐*OE* lines at 3.5 d. Scale bar, 1 cm. **(C**, **D)** Dynamic of germination rate among Nip and *ks1* mutant **(C)**, ZH11 and *OsGA2ox3*‐*OE* lines **(D)**. The data are shown as mean ± *SD*, *n* = 3 biological replicates.

OsKS1 is involved in an early step of the GA biosynthesis pathway and OsGA2ox3 is involved in the GA catabolic pathway. Knockout of *OsKS1* or overexpression of *OsGA2ox3* results in significantly lower levels of bioactive GAs (Sakamoto et al., 2004; Lo et al., 2008; Qin et al., 2022). To investigate whether OsKS1 and OsGA2ox3 regulate seed germination, we examined the seed germination rate of *ks1* mutant, *osga2ox3* mutant, and *OsGA2ox3*‐*OE* lines. The *ks1* (Li et al., 2020), *osga2ox3* mutant, and *OsGA2ox*‐*OE* lines (Qin et al., 2022) were previously described. The *ks1* mutant harbors a *Tos17* insertion in exon 3 of *OsKS1* (Figure S4A, B), *OsGA2ox3* was expressed at levels 17–20‐fold higher in the *OsGA2ox3*‐*OE* lines relative to their wild‐type control Zhonghua 11 (ZH11) (Figure S4C). The seed germination rates of the *ks1* mutant and *OsGA2ox3*‐*OE* lines were significantly lower than that of Nip or ZH11 over the time course from 0 to 5.5 d after imbibition (Figure 4), whereas the germination rates of the two *osga2ox3* mutants were identical to that of Nip (Figure S5A, B). The contents of GA precursors (GA_19_, GA_24_, GA_44_, and GA_53_) and bioactive GAs (GA_3_ and GA_4_) were similar between Nip and *osga2ox3* mutants (Figure S5C), suggesting functional redundancy among OsGA2ox members in seed germination and GA inactivation. Taken together, our results suggest that lower contents of bioactive GAs, caused by knocking out *OsKS1* or overexpressing *OsGA2ox3*, delayed seed germination.

### OsSLR1 physically interacts with OsABI5 to enhance its transcriptional activity

**Figure 5:**
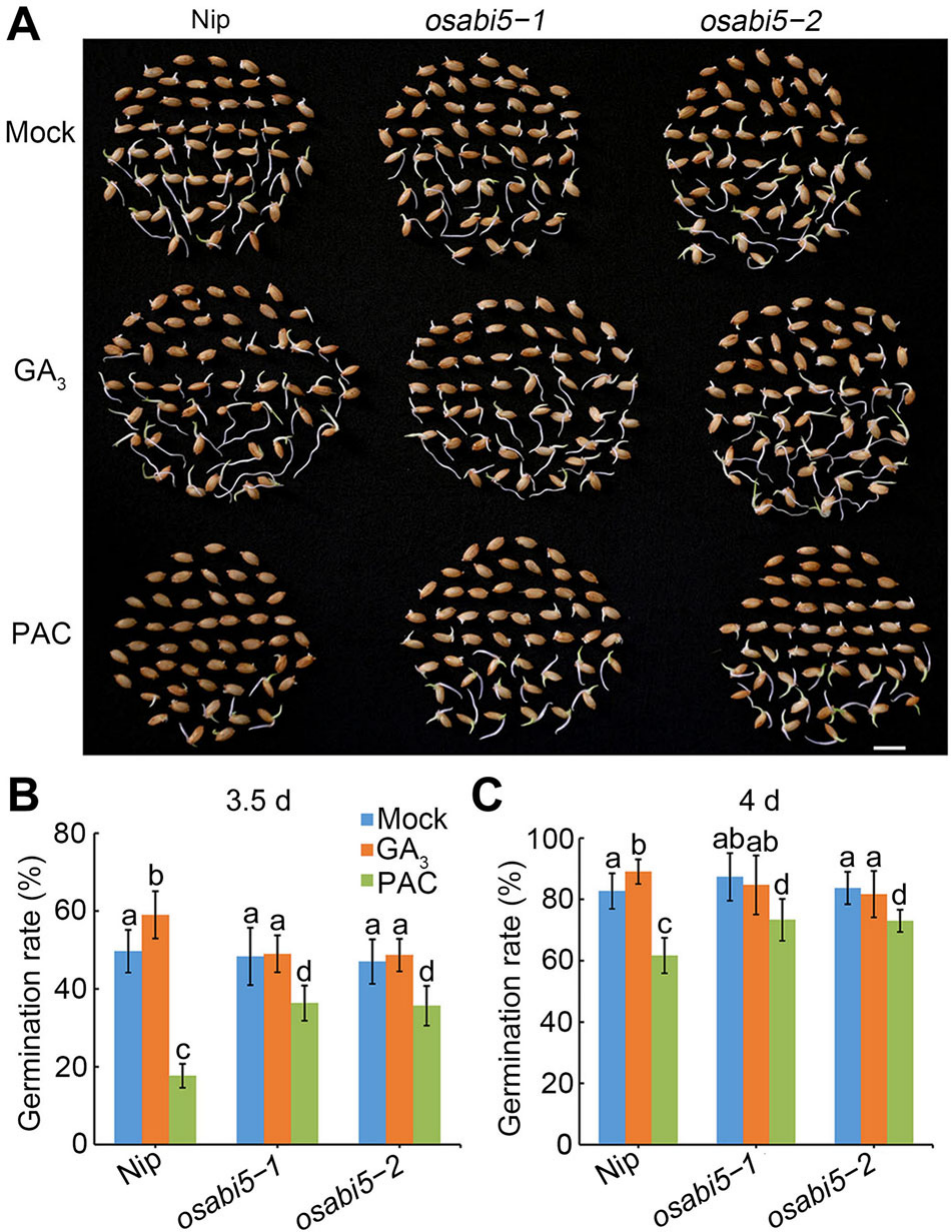
OsABI5 is required for GA‐promoted seed germination **(A)** Representative images of seed germination performance of Nipponbare (Nip) and *osabi5* mutants with or without 5 μmol/L GA_3_ or 2 μM paclobutrazol (PAC) treatment for 3.5 d. Scale bar, 1 cm. **(B**, **C)** Germination rate of seeds in **(A)** at 3.5 d **(B)** and 4 d **(C)**. The data are shown as mean ± *SD*, *n* = 3 biological replicates. Different letters indicate significant differences (*P* < 0.05, one‐way analysis of variance with Tukey's test).

Our previous study demonstrated that OsSAE1 directly represses *OsABI5* expression to promote seed germination (Li et al., 2022b). Numerous studies in Arabidopsis have shown that AtABI5 acts as a hub in the ABA–GA antagonism during seed germination (Liu and Hou, 2018; Li et al., 2022c; Xian et al., 2024). To investigate the role of OsABI5 in GA‐promoted seed germination, we treated seeds of two *osabi5* mutants with GA_3_ and PAC. GA_3_ treatment significantly promoted the germination of Nip seeds, but not those of the *osabi5* mutants (Figure 5). By contrast, PAC treatment significantly inhibited the germination of Nip seeds, as well as that of seeds from the *osabi5* mutants, although to a lesser extent (Figure 5). These results indicate that OsABI5 is required for GA‐induced seed germination.

**Figure 6:**
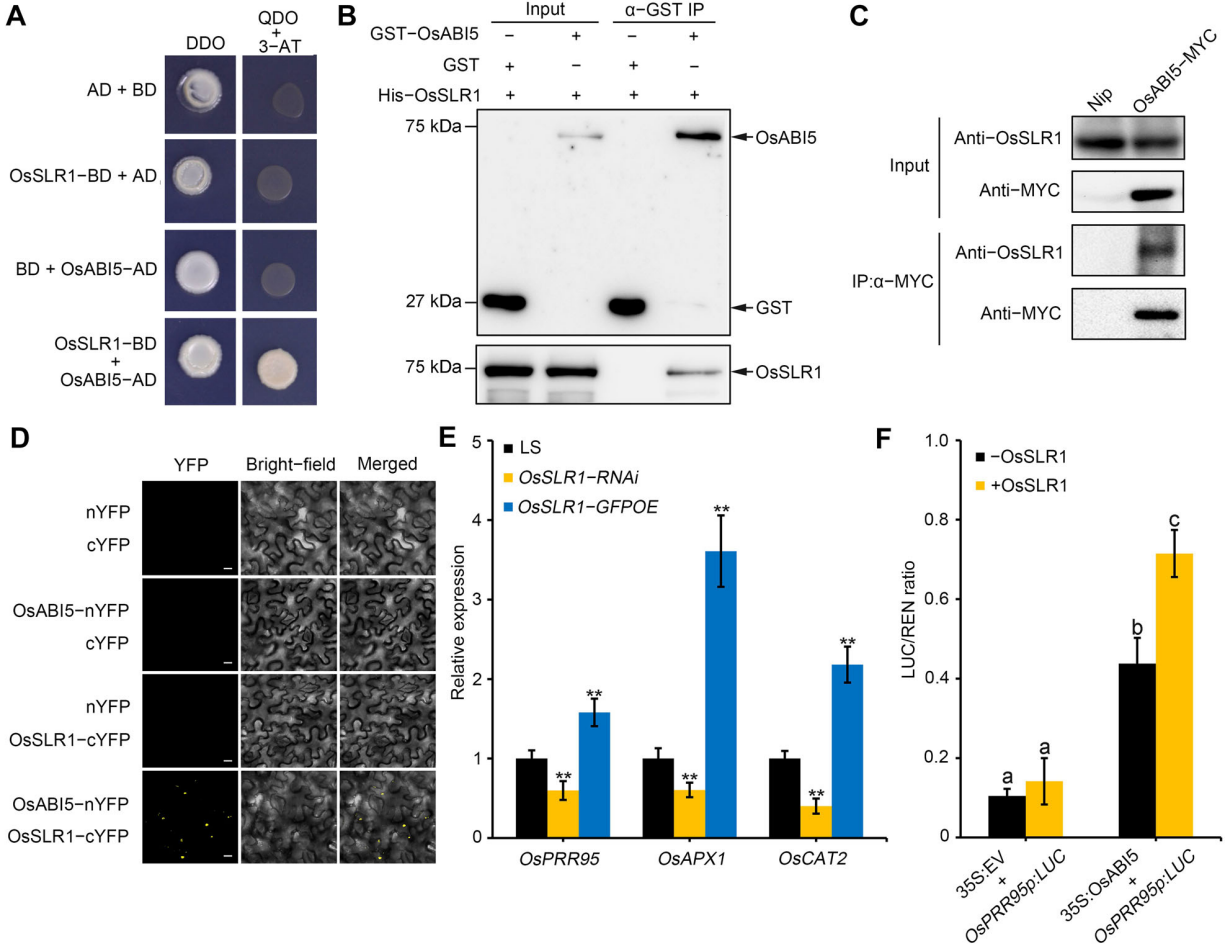
OsSLR1 directly interacts with OsABI5 to promote its transcriptional activation ability **(A)** Yeast‐two‐hybrid assay of OsSLR1 with OsABI5. Transformed yeast cells were grown for 3 d on selective medium SD/−Leu/−Trp/−His/−Ade (quadruple dropout (QDO)) containing 5 mmol/L 3‐AT and SD/−Trp/−Leu (DDO) medium. **(B)** *In vitro* pull‐down assays of the interaction between OsSLR1 and OsABI5. His‐OsSLR1 was incubated with glutathione S‐transferase (GST)‐OsABI5 or GST alone. After pull‐down with GST beads, the eluates were analyzed by immunoblots using the indicated antibodies. **(C)** Co‐immunoprecipitation (Co‐IP) assays of proteins isolated from OsABI5‐MYC and Nipponbare (Nip) plants by MYC antibody. **(D)** Bimolecular fluorescent complementation (BiFC) assay of OsSLR1 and OsABI5 co‐expressed in tobacco leaves. Scale bar, 20 μm. **(E)** Expression of *OsPPR95*, *OsAPX1* and *OsCAT2* in Lansheng (LS), *OsSLR1*‐*RNAi* and *OsSLR1*‐*GFPOE* lines. Seeds germinated for 2 d after imbibition were used for RNA extraction. *Actin1* was used as an internal control. The relative expression levels were represented by fold change relative to the expression levels of LS. Data are presented as mean ± *SD*, *n* = 3 biological replicates. Asterisks indicate significant differences compared with LS at **P < 0.01 (Student's *t*‐test). **(F)** Dual‐luciferase reporter assay showing that OsSLR1 increases OsABI5 transcriptional activity for its targets genes. The data are shown as mean ± *SD*, *n* = 3 biological replicates. Different letters indicate significant differences (*P* < 0.05, one‐way analysis of variance with Tukey's test).

OsSLR1 is the only DELLA protein in rice and DELLAs are co‐factors that regulate the activity of transcription factors in plants (Itoh et al., 2002; Mo et al., 2020; Xian et al., 2024). The requirement for OsABI5 in GA‐induced seed germination raised the possibility that OsSLR1 might directly interact with OsABI5. To test this hypothesis, we examined the germination rates of *OsSLR1*‐*RNAi* (knockdown of *OsSLR1* by RNA interference) and *OsSLR1*‐*GFPOE* (overexpressing *OsSLR1* fused to GFP) seeds. The germination rate of *OsSLR1*‐*RNAi* seeds was slightly higher, and that of *OsSLR1*‐*GFPOE* seeds was significantly lower, than that of Lansheng (LS) seeds (Figure S6), indicating that OsSLR1 is a negative regulator of seed germination. Application of GA_3_ largely rescued the lower seed germination rate of *OsSLR1*‐*GFPOE* seeds, whereas PAC treatment aggravated the already lower seed germination rate of *OsSLR1*‐*GFPOE* seeds (Figure S6). Moreover, the germination rate of *OsSLR1*‐*RNAi* seeds was less affected by GA_3_ and PAC treatment than that of LS seeds (Figure S6). These results indicate that GA promotes seed germination via its effects on DELLA protein OsSLR1. Subsequently, we performed multiple assays to test whether OsSLR1 interacts with OsABI5. Yeast two‐hybrid (Y2H) assays showed that OsSLR1 interacts with OsABI5 in yeast cells (Figure 6A). *In vitro* pull‐down assays using recombinant purified GST‐tagged OsABI5 and His‐tagged OsSLR1 confirmed that OsSLR1 interacts with OsABI5 (Figure 6B). Immunoblotting analysis with an anti‐OsSLR1 antibody in a co‐immunoprecipitation (Co‐IP) assay with an anti‐MYC antibody using protein extracts from plants expressing *OsABI5*−*MYC* revealed the presence of endogenous OsSLR1 in the co‐precipitates (Figure 6C). A bimolecular fluorescent complementation (BiFC) assay confirmed the interaction between OsSLR1 and OsABI5 (Figure 6D). Indeed, we detected fluorescence signal from reconstituted yellow fluorescent protein (YFP) in the nuclei of cells from *Nicotiana benthamiana* leaves expressing *OsABI5‐nYFP* and *OsSLR1‐cYFP*, but not from those of cells co‐expressing *OsABI5‐nYFP* and *cYFP*, or *OsSLR1‐cYFP* and *nYFP* (Figure 6D). These results demonstrate that OsABI5 physically interacts with OsSLR1, both *in vitro* and *in vivo*.

Next, we investigated the effect of OsSLR1 on the transcriptional activity of OsABI5. To rule out an influence of OsSLR1 on *OsABI5* expression, we measured *OsABI5* transcript levels in LS, *OsSLR1*‐*RNAi*, and *OsSLR1*‐*GFPOE* plants and found that the expression of *OsABI5* was not regulated by OsSLR1 (Figure S7A), indicating that *OsABI5* is not regulated by OsSLR1 at the transcriptional level. Previous studies have shown that OsABI5 directly activates the transcription of *CATALASE 2* (*OsCAT2*), *ASCORBATE PEROXIDASE 1* (*OsAPX1*), and *PSEUDO*‐*RESPONSE REGULATOR 95* (*OsPRR95*) to regulate seed germination in rice (Li et al., 2021, 2022a; Wang et al., 2023). Reverse transcription qPCR analysis revealed significantly higher *OsCAT2*, *OsAPX1*, and *OsPRR95* transcript levels in *OsSLR1*‐*GFPOE* seeds, and significantly lower transcript levels in *OsSLR1*‐*RNAi* seeds, compared with LS seeds (Figure 6E). Since the OsABI5−*OsAPX1* and OsABI5−*OsCAT2* modules mediate the stimulatory effects of melatonin on seed germination under low‐temperature and chromium stress (Li et al., 2021, 2022a), while the OsABI5−*OsPRR95* module mediates seed germination under normal growth conditions (Wang et al., 2023), we focused on *OsPRR95*. We employed a dual‐LUC reporter system, in which the *LUC* reporter gene was driven by the *OsPRR95* promoter. We co‐transfected rice protoplasts with the LUC reporter construct and the effector construct *35S:OsABI5* alone or together with *35S:OsSLR1*. The LUC activity derived from the *OsPRR95* promoter was strongly induced in the presence of OsABI5, and further enhanced in the presence of *35S:OsSLR1* (Figure 6F), suggesting that OsSLR1 significantly promotes the transcriptional activity of OsABI5 toward its target genes. When we performed a tsCUT&Tag‐qPCR assay using genomic DNA extracted from ZH11 and *OsSLR1‐OE* protoplasts transfected with OsABI5‐GFP, we found no significant difference in OsABI5‐GFP binding to the *OsPRR95* promoter between *OsSLR1‐OE* and ZH11 protoplasts (Figure S7B), suggesting that the physical interaction between OsSLR1 and OsABI5 does not affect OsABI5 binding to its target genes.

### Genetic interaction of *OsSLR1* and *OsABI5*

**Figure 7:**
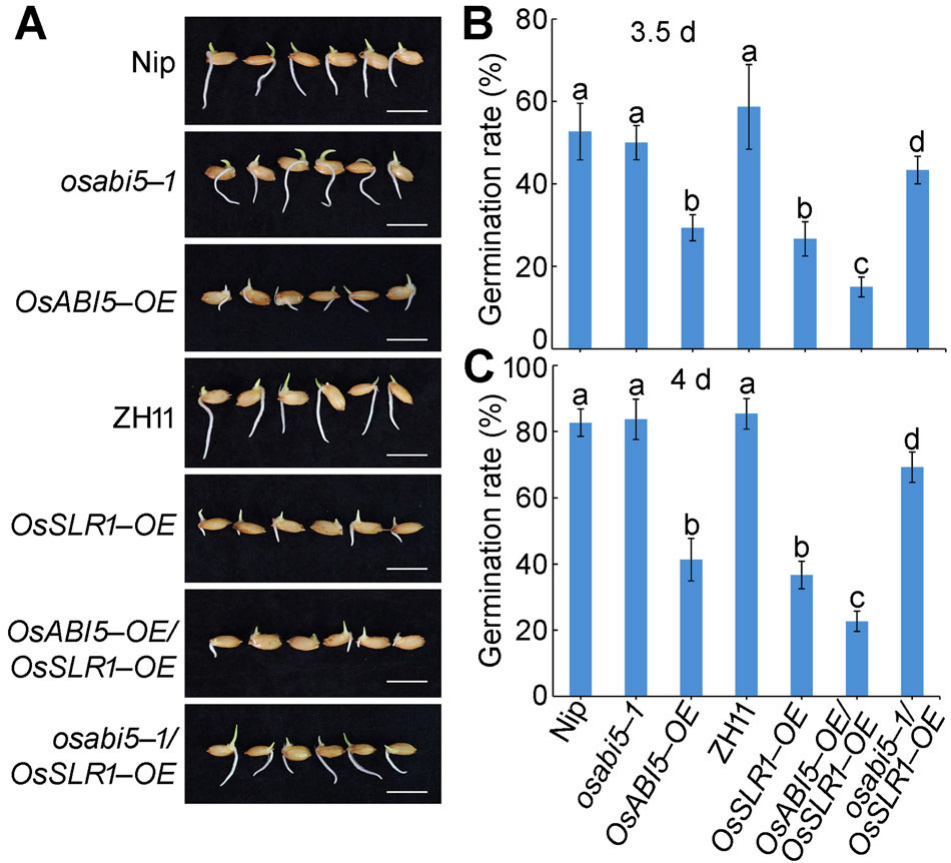
OsABI5 acts downstream of OsSLR1 to regulate seed germination **(A)** Representative images of seed germination performance of Nipponbare (Nip), *osabi5*‐*1*, *OsABI5*‐*OE*, ZH11, *OsSLR1*‐*OE*, *OsABI5*‐*OE OsSLR1*‐*OE*, and *osabi5 OsSLR1*‐*OE* at 3.5 d. Scale bar, 1 cm. **(B**, **C)** Germination rate of seeds in **(A)** at 3.5 d **(B)** and 4 d **(C)**. The data are shown as mean ± *SD*, *n* = 3 biological replicates. Different letters indicate significant differences (*P* < 0.05, one‐way analysis of variance with Tukey's test).

To study the genetic relationship between *OsSLR1* and *OsABI5*, we analyzed the germination rate of seeds from a *OsABI5*‐*OE OsSLR1*‐*OE* line obtained by crossing *OsABI5*‐*OE* to *OsSLR1*‐*OE*. The germination rate of *OsABI5*‐*OE OsSLR1*‐*OE* seeds was lower than that of *OsABI5*‐*OE* and *OsSLR1*‐*OE* seeds (Figure 7), suggesting a synergistic interaction between *OsSLR1* and *OsABI5* to regulate seed germination.

To further examine the genetic relationship between *OsSLR1* and *OsABI5*, we generated an *osabi5*‐*1 OsSLR1*‐*OE* line by crossing the *osabi5*‐*1* mutant to an *OsSLR1*‐*OE* line. The germination rate of *osabi5*‐*1 OsSLR1*‐*OE* seeds was significantly higher than that of *OsSLR1*‐*OE* seeds but slightly lower than that of *osabi5*‐*1* seeds (Figure 7). These data suggest that *OsABI5* acts downstream of *OsSLR1* and that the pathway mediated by OsABI5 is in part required for OsSLR1 signaling during the regulation of seed germination.

### Temporal expression of *OsSAE1* orchestrates seed germination

**Figure 8:**
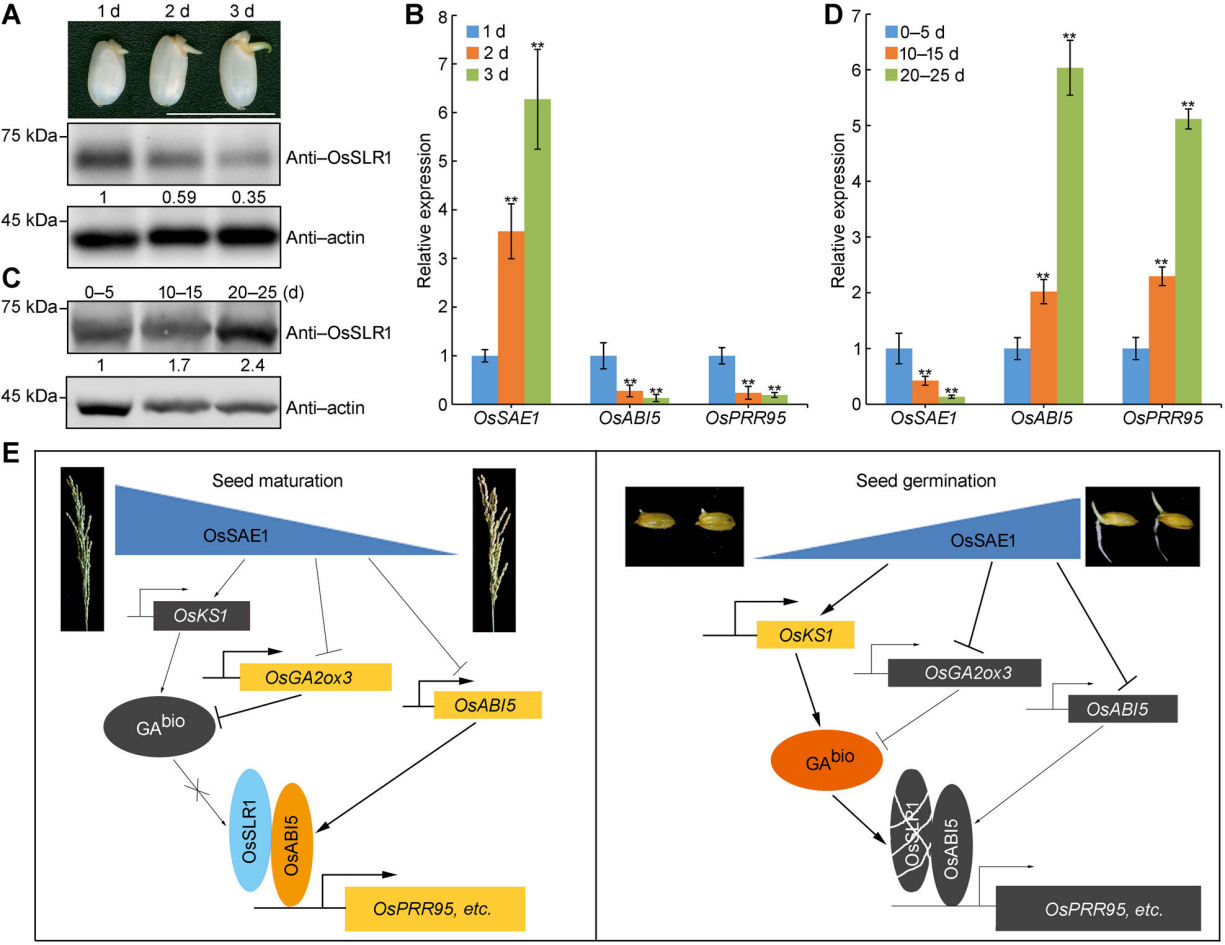
Temporal expression of *OsSAE1* controls seed germination and seed dormancy in rice **(A**, **C)** SLENDER RICE1 (OsSLR1) protein levels during seed germination **(A)** and seed maturation **(C)**. Total proteins extracted from Nipponbare (Nip) seeds harvested at different days after imbibition **(A)** or pollination **(C)** was analyzed by immunoblotting. Anti‐OsSLR1 antibody was used to detect protein levels of OsSLR1. Actin was used as a loading control. **(B**, **D)** Expression of *OsSAE1*, *OsABI5* and *OsPPR95* during seed germination **(B)** and seed maturation **(D)**. *Actin1* was used as an internal control. The relative expression levels are represented by fold change relative to the expression levels of 1 d **(B)** or 0–5 d **(D)**. Data are presented as mean ± *SD*, *n* = 3 biological replicates. Asterisks indicate significant differences compared with 1 d **(B)** or 0–5 d **(D)** at **P < 0.01 (Student's *t*‐test). **(E)** Schematic representation of OsSAE1 integrates abscisic acid (ABA) and gibberellin (GA) signaling to regulate seed germination and seed dormancy. The expression of *OsSAE1* decreased during seed maturation and increased during seed germination. Enhanced expression of *OsSAE1* directly repressed the expression of *OsABI5* to promote seed germination. In parallel, OsSAE1 directly regulates the expression of *OsKS1* and *OsGA2ox3* to promote the accumulation of bioactive GAs, leading to the degradation of OsSLR1 to weaken the transcriptional activation ability of OsABI5 toward its target genes, ultimately promoting seed germination. The arrows indicate stimulatory effects, whereas the T sharp symbol indicates inhibitory effects, and the thickness of the lines indicates the strength of regulation.

The ABA/GA ratio in seeds dictates seed dormancy and germination (Shu et al., 2016a). The involvement of OsSAE1 in ABA−GA antagonism prompted us to explore how OsSAE1 balances GA and ABA signaling to modulate seed germination in rice. To address this question, we examined the abundance of OsSLR1 over the course of seed germination by immunoblotting with an anti‐OsSLR1 antibody. OsSLR1 abundance gradually declined during seed germination (Figure 8A), while the expression levels of *OsSAE1* gradually rose, in contrast to the diminishing expression of *OsABI5* and *OsPRR95* (Figure 8B).

Proper seed dormancy ensures that seeds do not germinate too early, a phenomenon known as preharvest sprouting, which negatively affects crop yield and grain quality (Sohn et al., 2021). We thus explored the possible function of OsSAE1 in seed dormancy by testing the germination rate of freshly harvested mature panicles. Seed from freshly harvested mature panicles of the two *ossae1* mutants only reached a germination rate of 9%, whereas the seed germination rate for the two *OsSAE1‐OE* lines was 57%, and that of Nip seeds was 42% at 7 d imbibition (Figure S8), indicating that OsSAE1 functions as a negative regulator of seed dormancy. In agreement with this result, OsSLR1 abundance and the expression levels of *OsABI5* and *OsPRR95* gradually increased during seed development, whereas *OsSAE1* expression gradually decreased (Figure 8C, D). Therefore, our results suggest that the temporal expression of *OsSAE1* orchestrates GA and ABA signaling to modulate seed germination and seed dormancy (Figure 8E).

### A superior *OsSAE1* allele improves seed germination

**Figure 9:**
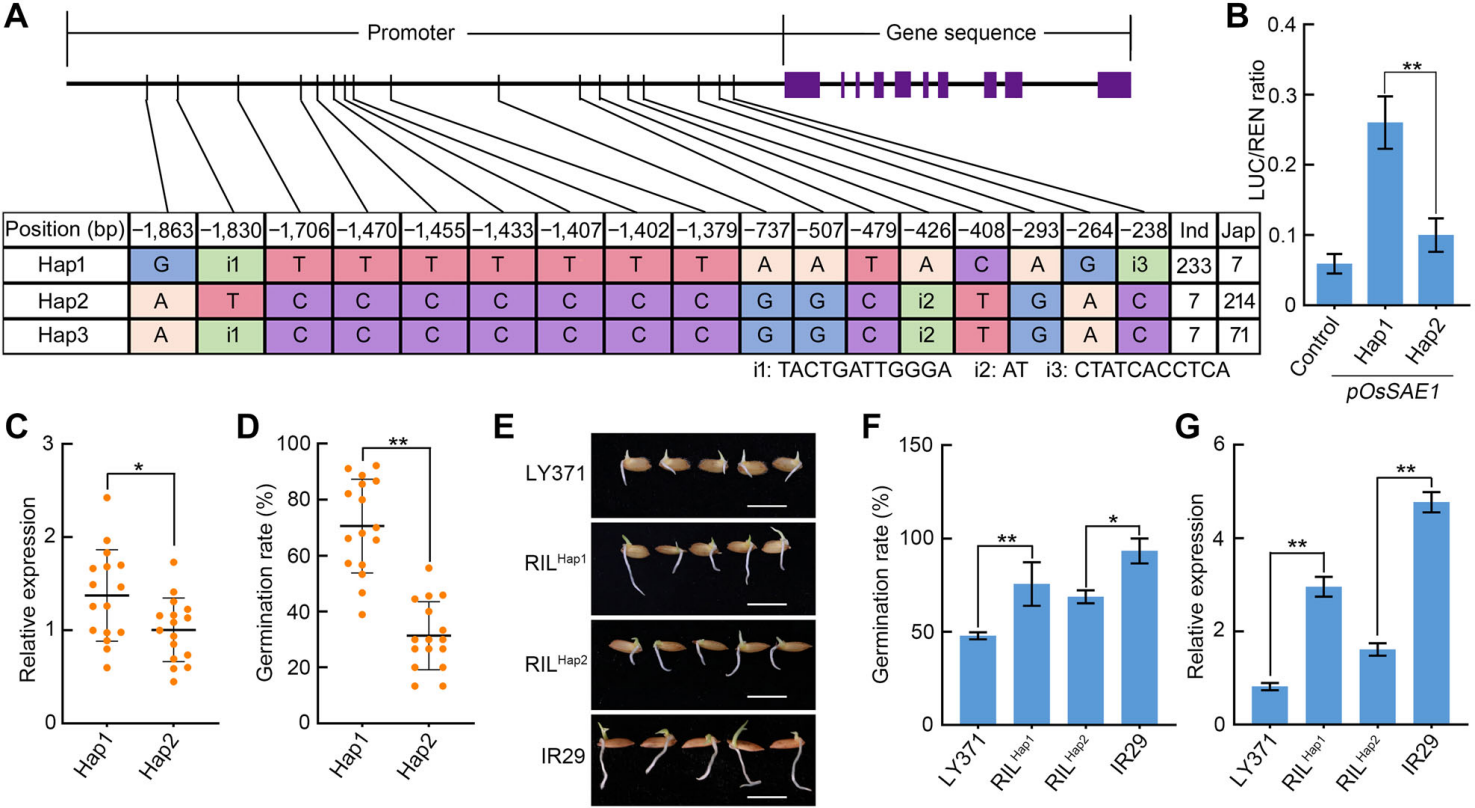
Elite allele of *OsSAE1* enhances seed germination in rice **(A)** Haplotype analysis of the *OsSAE1* promoter region in 578 rice cultivars. **(B)** Transient promoter activity of the haplotypes (*pOsSAE1* ^*Hap1*^ and *pOsSAE1* ^*Hap2*^) of *OsSAE1* promoters. Data were presented as mean ± *SD*, *n* = 3 biological replicates. **(C)** Expression of *OsSAE1* in the germinating (2 d after imbibition) seeds in rice accessions containing the different haplotypes (Hap1 and Hap2). Center lines show the medians. *n* = 16 accessions. **(D)** Germination rate of seeds in **(C)** at 4 d. Center lines show the medians. *n* = 16 accessions. **(E)** Representative images of seed germination performance of Liaoyan 371 (LY371), RIL^Hap1^, RIL^Hap2^, and IR29 at 3 d. Scale bar, 1 cm. **(F)** Germination rate of seeds in **(E)** at 4 d. Data are presented as mean ± *SD*, *n* = 3 biological replicates. **(G)** Expression of *OsSAE1* in the germinating (2 d after imbibition) seeds in LY371, RIL^Hap1^, RIL^Hap2^, and IR29. Data are presented as mean ± *SD*, *n* = 3 biological replicates. For **(B–D**, **F** and **G)**, asterisks indicate significant differences between the two compared samples using a Student's *t*‐test at *P < 0.05 and **P < 0.01.

To identify a favorable *OsSAE1* allele that would confer improved seed germination, we analyzed the nucleotide polymorphisms in the coding region and 2,000 bp region upstream of *OsSAE1* across 578 rice accessions (Quan et al., 2024). We identified three major *OsSAE1* haplotypes (Hap1, Hap2, and Hap3) in these accessions (Figures 9A, S9A). Notably, Hap1 was predominant in the *indica* cultivars, while Hap2 and Hap3 were primarily found in the *Japonica* cultivars (Figures 9A, S9A), suggesting an *indica*–*japonica* differentiation among the *OsSAE1* haplotypes.

To investigate whether natural variation in the *OsSAE1* promoter or coding region affects seed germination, we performed a dual‐LUC reporter activity assay in rice protoplasts (Figures 9B, S9B−D). Since Hap2 and Hap3 of *OsSAE1* only differ at one site each in the promoter (−1,830) and coding region (+3,119), and are primarily derived from *Japonica* cultivars (Figures 9A, S9A), we focused on Hap1 and Hap2 for further analysis. Our results showed there were no significant differences in the transactivation activity of the LUC reporter gene between *OsSAE1* ^*Hap1*^ and *OsSAE1* ^*Hap2*^ containing different coding regions (Figure S9B, C). Further examination of the transcriptional activation activity of OsSAE1^Hap1^ and OsSAE1^Hap2^ on the downstream genes *OsGA2ox3* and *OsKS1* found that there were no significant differences in the transactivation activity of OsSAE1^Hap1^ and OsSAE1^Hap2^ on these two downstream genes (Figure S9D), indicating that natural variation in the *OsSAE1* coding region does not affect its transcriptional activation activity. However, the *pOsSAE1* ^*Hap1*^ promoter showed a much higher LUC activity than the *pOsSAE1* ^*Hap2*^ (Figure 9B), suggesting that OsSAE1 expression may vary among rice accessions as a function of the polymorphisms in its promoter. We tested this hypothesis by measuring *OsSAE1* transcript levels in 32 rice cultivars (16 cultivars carrying Hap1 and 16 cultivars harboring Hap2), which revealed that *OsSAE1* expression levels are significantly higher in cultivars possessing Hap1 than in those with Hap2 (Figure 9C). These differences in *OsSAE1* expression were reflected in the germination rates, with significantly higher germination rates for the cultivars carrying Hap1 than those with Hap2 (Figure 9D). These results suggest that Hap1 is a superior haplotype associated with high seed germination; importantly, this allele has not been well exploited in breeding programs for *japonica* varieties.

We generated recombinant inbred lines (RILs) by crossing IR29 (high seed germination rate, harboring Hap1) to Liaoyan 371 (LY371, low seed germination rate; carrying Hap2). We selected RILs with *OsSAE1* ^*Hap1*^ in an otherwise LY371 background and RILs with *OsSAE1* ^*Hap2*^ in an otherwise IR29 background. The RILs with *OsSAE1* ^*Hap1*^ exhibited an improved seed germination over that of LY371, whereas the RILs with *OsSAE1* ^*Hap2*^ showed a lower seed germination rate than IR29 (Figure 9E, F). Consistent with this result, *OsSAE1* transcript levels were higher in those RILs harboring *OsSAE1* ^*Hap1*^ in the LY371 background and lower in RILs carrying *OsSAE1* ^*Hap2*^ in the IR29 background (Figure 9G). These results suggest *OsSAE1* as a potential target for breeding rice cultivars with enhanced seed germination for direct seeding.

## DISCUSSION

Seed germination is a crucial event in the life of land plants to establish the next generation and is controlled by interactions among endogenous developmental signals, especially the phytohormones ABA and GA (Shu et al., 2016a; Lu et al., 2023; Huang et al., 2024). In our previous study, we demonstrated that the AP2/ERF transcription factor OsSAE1 positively regulates rice seed germination by modulating the *OsABI5*‐mediated pathway (Li et al., 2022b). Here, we demonstrated that OsSAE1 orchestrates the antagonistic regulation of ABA and GA signaling during rice seed germination. OsSAE1 directly activated the expression of the GA biosynthesis gene *OsKS1* and repressed that of the GA metabolism gene *OsGA2ox3* to promote the accumulation of bioactive GAs, leading to the degradation of OsSLR1 and weakening the transcriptional activation ability of OsABI5 toward its target genes, ultimately promoting seed germination. Collectively, our data reveal that OsSAE1 acts as a switch that balances the antagonistic effects of ABA and GA during seed germination.

As the primary phytohormones regulating seed germination and seed dormancy, ABA and GA antagonistically mediate diverse aspects of plant development (Liao et al., 2023; Xian et al., 2024; Xie et al., 2024), thus the homeostasis between ABA and GA is essential for plant growth and development. Several regulators involved in this ABA−GA antagonism have been reported, such as AtABI4 (Shu et al., 2016b), AtDDF1 (Magome et al., 2008), and AtPER1 (Chen et al., 2020) in Arabidopsis, or OsAP2‐39 (Yaish et al., 2010) and OsNAC120 (Xie et al., 2024) in rice. However, our understanding of the precise mechanisms underlying the ABA−GA antagonism is far from complete. In the present study, we showed that the OsSLR1−OsABI5 module is required for OsSAE1‐regulated seed germination in rice. OsSLR1 directly interacted with OsABI5 to enhance its transcriptional activation ability and prevent seed germination. Mutation of *OsABI5* attenuated GA‐promoted seed germination, and the low germination rate of *OsSLR1*‐*OE* seeds was largely rescued to wild‐type levels by knocking out *OsABI5* in this background. Overexpression of *OsSAE1* resulted in the accumulation of bioactive GAs, which in turn promote the degradation of OsSLR1, thereby promoting seed germination. Thus, our results uncover an OsSAE1‐based regulatory network of ABA−GA antagonism, deepening our understanding of the antagonism between these two phytohormones.

Rice is a staple food for more than half of the global population. Direct rice seeding is an increasingly popular low‐cost and convenient practice compared with conventional transplantation practices (Farooq et al., 2011). However, direct seeding is constrained by seed vigor under different stress conditions in the wild. Thus, identifying genes involved in seed vigor and dissecting their underlying mechanisms should facilitate the breeding of rice cultivars amenable to direct seeding. In this study, we demonstrated that OsSAE1 positively regulates seed germination by modulating the ABA−GA antagonism. Therefore, OsSAE1 represents a potential target for breeding rice cultivars with high seed germination rates suitable for direct seeding. Notably, enhanced expression of *OsSAE1* weakened seed dormancy. Weak seed dormancy leads to unwanted early sprouting of freshly matured seeds still encased in the spikes of the mother plant, thus diminishing grain quality and yield in rice (Tai et al., 2021; Xu et al., 2022; Fan et al., 2024). Therefore, the precise manipulation of *OsSAE1* expression might allow seed germination in a timely manner. Using specific promoters, such as tissue‐specific promoters or inducible promoters, to optimize *OsSAE1* expression in a precise temporal and spatial manner would help strike the right balance between seed dormancy and seed germination. In addition, *OsSAE1* expression gradually increased during seed germination, but decreased during seed maturation, suggesting that *OsSAE1* expression is already highly regulated. Identification of the *cis*‐acting elements and transcription factors associated with *OsSAE1* transcription will offer targets for genetic manipulation via clustered regularly inter‐spaced short palindromic repeats (CRISPR)‐associated protein 9 (Cas9)‐mediated editing, and will reveal insight into how plants integrate phytohormonal signals to regulate seed germination and dormancy.

Mounting evidence highlights the value of natural variation as a resource for genetic improvement of agronomic traits through plant breeding (Liang et al., 2021). Rice cultivated in Asia comprises two main subspecies, *indica* and *japonica*, which differ in various developmental and physiological traits (Sun et al., 2023). Previous studies revealed a tendency toward higher seed vigor in *indica* varieties than in *japonica* varieties (Peng et al., 2022). However, the underlying mechanism is largely unclear. Our findings indicate that natural variation in the *OsSAE1* promoter contributes to variation in seed germination by influencing *OsSAE1* expression. Indeed, cultivars harboring the superior Hap1 allele of *OsSAE1* had higher seed germination rates than those carrying Hap2. The Hap1 allele is mainly present in *indica* cultivars, suggesting that *OsSAE1* may have been selected during the *japonica*–*indica* differentiation. Recombinant inbred lines carrying the superior Hap1 allele of *OsSAE1* in an otherwise *japonica* background had significantly higher seed germination rates than the parental *japonica* variety with Hap2. These results strongly support the notion that Hap1 of *OsSAE1* is a candidate for molecular breeding to improve seed germination in rice, via marker‐assisted selection of Hap1 or overexpression of *OsSAE1* ^*Hap1*^.

We previously demonstrated that OsSAE1 binds directly to the *OsABI5* promoter to repress its transcription and control seed germination (Li et al., 2022b). In this study, we provide evidence that OsSAE1 regulates rice seed germination by mediating the antagonism between ABA and GA signaling. On the basis of our present and previous data, we propose a modulatory model by which OsSAE1 orchestrates seed germination (Figure 8E). *OsSAE1* expression is precisely regulated during seed maturation and germination. OsSAE1 directly represses *OsABI5* expression while at the same time promoting the degradation of OsSLR1 to attenuate the transcriptional activation ability of OsABI5 toward its target genes by activating GA biosynthesis and repressing GA catabolism, ultimately accelerating seed germination. Our findings deepen our understanding of the mechanisms underlying ABA–GA antagonism, which will be important for expanding the cultivation of rice through direct seeding.

## MATERIALS AND METHODS

### Plant materials and grown conditions

The rice genotypes used in this study were *ossae1*, *OsSAE1*‐*OE*, *ks1*, *OsGA2ox3*‐*OE*, *osga2ox3*, *osabi5*, *OsABI5*‐*OE*, *OsSLR1*‐*RNAi*, *OsSLR1*‐*GFPOE*, and *OsSLR1*‐*OE* and were previously described (Liao et al., 2019; Li et al., 2020, 2022b, 2023; Qin et al., 2022). The backgrounds for the various lines are as follows: *ossae1*, *OsSAE1*‐*OE*, *ks1*, *osga2ox3*, *osabi5*, and *OsABI5*‐*OE* in Nipponbare (Nip) (*Oryza sativa*, *Japonica*); *OsGA2ox3*‐*OE*, *OsSLR1*‐*OE* in Zhonghua 11 (ZH11) (*Oryza sativa*, *Japonica*); *OsSLR1*‐*RNAi*, *OsSLR1*‐*GFPOE* in Lansheng (LS) (*Oryza sativa*, *Japonica*). For the construction of RIL‐*OsSAE1*‐Hap1 and RIL‐*OsSAE1*‐Hap2, the *indica* variety IR29 was crossed to the *japonica* variety Liaoyan 371 (LY371). F6 generation progeny homozygous for *OsSAE1*‐Hap1 or *OsSAE1*‐Hap2 were used for seed germination assays. For material propagation and cross, rice plants were cultivated in the experimental field of the Chinese Academy of Agricultural Sciences in Langfang (39°52′ N, 116°70′ E) from May to October each year.

### Seed germination assays

For seed germination assay, 50 freshly harvested and dried seeds per replicate were imbibed in Petri dishes containing 10 mL sterile distilled water and incubated at 4°C for 48 h. Water was removed by draining and replaced with a 5 μmol/L GA_3_ or 2 μmol/L PAC solution before the plates were placed in a growth chamber under a 14‐h light/10‐h dark photoperiod at 28°C. Germination was defined as radical emergence, and germinated seeds were scored every 12 h. The germination rate was calculated as the number of germinated seeds divided by 50 (total number of seeds assayed). Three replicates were performed for each experiment.

### Reverse transcription qPCR

Total RNA was extracted from seeds using a Plant Total RNA Purification Kit (GeneMark, Taichung, Taiwan) or from other tissues using an Ultrapure RNA Kit (CWBIO, jiangsu, China) before being subjected to reverse transcription using a HiScript II Q RT SuperMix Kit (Vazyme, Nanjing, China) according to the manufacturer's instructions. Quantitative PCR was performed as previously described (Zhang et al., 2012), using the *OsACTIN1* gene to normalize gene expression. Primers used for RT‐qPCR are listed in Table S1.

### Qualitative and quantitative analyses of seed α‐amylase activity

To qualitatively evaluate α‐amylase activity in Nip, *ossae1*, and *OsSAE1*‐*OE* seeds during germination, a starch plate test assay was conducted following the method of Xiong et al. (2022) with minor modifications. Briefly, rice seeds were surface‐sterilized and transversely cut in half. The half without an embryo was transferred to plates containing 0.2% (w/v) soluble starch with the cut face down in tight contact with the medium surface. After incubation at 28°C for 48 h in the dark, the plate was soaked in iodine solution containing 0.1% (w/v) I_2_ and 1% (w/v) KI for 5 min. Starch hydrolysis by α‐amylase results in a colorless halo. Quantification of α‐amylase activity was carried out using a colorimetric α‐amylase assay kit (Solarbio, Beijing, China) according to the manufacturer's instructions.

### Transient and simplified CUT&Tag assay and analysis

The tsCUT&Tag assay was performed as previously described (Wu et al., 2022a). The full‐length coding sequence of *OsSAE1* or *OsABI5* was cloned in‐frame and upstream of the enhanced GFP‐coding sequence in the pAN580 vector under the control of the *35S* promoter. The resulting constructs were individually transfected into rice protoplasts, followed by incubation at 28°C for 16 h in the dark. The empty pAN580 vector was transfected as a negative control. The CUT&Tag assay was performed using a Hyperactive Universal CUT&Tag Assay Kit (Vazyme, Nanjing, China) according to the manufacturer's instructions. Briefly, ~10^5^ successfully transfected live protoplasts were collected, immediately immobilized on ConA MagPloy Beads, and lysed with digitonin to allow binding of primary and secondary antibodies and the core hyperactive pA/G‐Tn5 transposase. A commercial mouse anti‐GFP antibody (TransGen, Beijing, China) was used as primary antibody (1:50 dilution, v/v) and incubated with the cell lysates for at least 2 h at room temperature or at 4°C overnight. The secondary antibody (1:100 dilution, v/v; Vazyme, Nanjing, China) was diluted in wash buffer and added to the mixture for a 1 h incubation at room temperature. The core hyperactive pA/G‐Tn5 transposase (0.04 μmol/L) loaded with mosaic‐end adapters was added to the antibody−protein complex and incubated at room temperature for 1 h. Tn5 was activated by addition of 10 mmol/L Mg^2+^ and incubation at 37°C for 1 h. DNA was extracted using the phenol‐chloroform method. After direct PCR amplification (15−20 cycles), the transcription factor−target DNA library was successfully constructed for sequencing. Quantitative PCR was performed using 1 μL purified DNA as template and was performed in triplicate for each target gene. The gene‐specific qPCR primers are listed in Table S1.

### Transactivation activity assays

To test the transactivation potential of OsSAE1, the full‐length *OsSAE1* coding sequence was cloned into the pGBKT7 vector harboring the GAL4 DNA‐binding domain (BD) to generate the OsSAE1‐BD plasmid. The construct was introduced into yeast strain Y2H Gold. Positive transformants were selected for growth on selective defined (SD) medium lacking tryptophan (SD/−Trp). Positive colonies were spotted onto SD/−Trp medium, SD/−Trp medium containing 0.3 μg/mL aureobasidin A (AbA), and SD/−Trp medium containing 5‐bromo‐4‐chloro‐3‐indoxyl‐α‐D‐galactopyranoside (X‐α‐gal).

A dual‐LUC reporter assay was conducted to test the transcriptional repression activity of OsSAE1. The full‐length *OsSAE1* coding sequence cloned in‐frame and downstream of the sequence encoding the transactivation domain from VP16 was fused with the GAL4 DBD to generate the pBD‐VP16‐OsSAE1 effector plasmid. The GAL4 DBD with or without VP16 (pBD and pBD‐VP16) was used as a negative and positive control, respectively. All the primers used are listed in Table S1.

### Yeast two‐hybrid assay

The full‐length coding sequences of *OsABI5* and *OsSLR1* were individually cloned into the pGADT7 or pGBKT7 vector to generate the OsABI5‐AD and OsSLR1‐BD plasmids, respectively. OsSLR1‐BD was co‐introduced into yeast strain AH109 with the OsABI5‐AD construct. The empty vectors were co‐introduced as a negative control. Positive transformants were selected for growth on SD/−Leu/−Trp medium, and positive colonies were spotted onto fresh SD/−Ade/−His/−Leu/−Trp (quadruple dropout (QDO)) medium containing 5 mmol/L 3‐AT according to the manufacturer's protocol (Clontech, Mountain View, CA, USA). All the primers used are listed in Table S1.

### *In vitro* pull‐down assay

The full‐length coding sequences of *OsABI5* and *OsSLR1* were cloned individually into the pET30a or pGEX6p‐1 vector to generate the GST‐OsABI5 and His‐OsSLR1 plasmids, respectively, for protein production. Recombinant GST‐OsABI5 and His‐OsSLR1 were purified using nickel and glutathione beads respectively (TransGen, Beijing, China). The two purified proteins were incubated at 4°C for 3 h, followed by pull‐down with the appropriate beads. Protein eluates were detected via immunoblotting with anti‐His and anti‐GST antibodies (Abmart, Shanghai, China). All the primers used are listed in Table S1.

### Bimolecular fluorescence complementation assay

The full‐length coding sequences of *OsABI5* and *OsSLR1* were cloned individually into the pSCYCE and pSCYNE vectors, respectively (Waadt et al., 2008). The resulting plasmids were introduced into Agrobacterium (*Agrobacterium tumefaciens*) strain GV3101. Positive Agrobacterium colonies carrying each construct of interest were co‐infiltrated into the abaxial side of leaves from 4‐week‐old *Nicotiana benthamiana* plants. The infiltrated plants were grown for 2 d before examination. Yellow fluorescent protein signals were observed with a confocal laser‐scanning microscope (LSM980; ZEISS). All the primers used are listed in Table S1.

### Co‐immunoprecipitation assay

For Co‐IP assay of OsABI5 by OsSLR1, total proteins were extracted from 2‐d‐old ABI5‐MYC or Nip seedlings in IP buffer containing 50 mmol/L Tris‐HCl (pH 7.5), 150 mmol/L NaCl, 5 mmol/L ethylenediaminetetraacetic acid (EDTA), 0.1% (v/v) NP‐40, 5 mmol/L dithiothreitol (DTT), 5% (v/v) glycerol, 1 mmol/L freshly added phenylmethylsulfonyl fluoride (PMSF), and 1× protease inhibitor cocktail. The protein mixture was incubated with magnetic MYC‐agarose beads (Chromo Tek, Planegg‐Martinsried, Germany) at 4°C for 2 h, followed by five washes with wash buffer containing 50 mmol/L Tris‐HCl (pH 7.5), 150 mmol/L NaCl, and 0.1% (v/v) NP‐40. The eluted immunoprecipitates were probed by immunoblotting with anti‐MYC (Abmart, Shanghai, China) and anti‐OsSLR1 antibodies.

### Dual‐LUC reporter assay

A 2.0‐kb promoter fragment of *OsKS1*, a 2.6‐kb promoter fragment of *OsGA2ox3*, and a 2.0‐kb promoter fragment of *OsPRR95* were individually amplified from genomic DNA using specific primers (Table S1) and cloned upstream of the *Firefly luciferase* (*LUC*) gene into the pGreen II 0800‐LUC vector, which also carries *Renilla luciferase* (*REN*) driven by the *35S* promoter as an internal reference for transfection efficiency. The effector constructs *35S:OsSAE1*, *35S:OsSLR1*, or *35S:OsABI5* and reporters were co‐transfected into rice protoplasts via the polyethylene glycol‐mediated method as previously described (Bart et al., 2006).

To test the transcriptional activation of the *OsSAE1* promoter, a 2.0‐kb *OsSAE1* promoter fragment was cloned into the pGreen II 0800‐LUC vector to generate the *proOsSAE1:LUC* plasmid, which was transfected into rice protoplasts as above. After 12 h of incubation in the dark, the protoplasts were collected by centrifugation and immediately assayed for LUC and REN activity. The pGreen II 0800‐LUC empty vector was used as a control.

Luciferase and REN activities were measured with a dual‐LUC reporting assay kit (Promega, Madison, WI, USA). Luciferase activity was normalized to REN activity, resulting in LUC/REN ratios. For each plasmid combination, three independent transfections were performed.

### Electrophoretic mobility shift assays

The full‐length coding sequence of *OsSAE1* was inserted into the pGEX6p‐1 vector, resulting in the GST‐OsSAE1 plasmid. Recombinant protein production was performed in *Escherichia coli* BL21 (DE3). Recombinant GST‐OsSAE1 was purified using glutathione beads (TransGen, Beijing, China). Electrophoretic mobility shift assays were performed *in vitro* using a LightShift Chemiluminescent EMSA Kit (Thermo Fisher, Waltham, MA, USA) according to the manufacturer's instructions. Unlabeled probes were synthesized and added to the reaction as cold competitors. All primers used in the EMSAs are listed in Table S1.

### Quantification of GA levels

To measure GA levels, seeds were harvested and the embryo dissected before detection of GA_3_, GA_4_, GA_19_, GA_24_, GA_44_, and GA_53_ levels. Briefly, seeds with the embryo were ground into powder. A 50 mg aliquot of powder was extracted with 1 mL methanol/H_2_O/formic acid (15:4:1, v/v/v), after which a 10 μL internal standard solution (10 ng/mL) was added for quantification. The mixture was vortexed for 15 min, and centrifugated at 4°C for 10 min at 13,000 *g*. The supernatant was transferred to clean plastic microtubes and evaporated to dryness. Then, 500 μL H_2_O containing 3.5% (v/v) formic acid and 1 mL ethyl acetate was added to the residue and the sample was vortexed for 15 min for resuspension. The sample was centrifugated at 4°C for 5 min at 13,000 *g*, and the supernatant was transferred to a brown injection vial. Then, 500 μL ethyl acetate was added to the previous tube containing the residue and centrifugated at 4°C for 5 min at 13,000 *g*. The supernatants were combined in the brown injection vial. The combined supernatant was evaporated to dryness and dissolved in acetonitrile. To the resulting solution, 10 μL triethylamine and 10 μL 3‐bromopropyltrimethylammonium bromide were added. The mixture was vortexed, incubated at 90°C for 1 h, and evaporated to dryness under a nitrogen gas stream, followed by resuspension in 100 μL acetonitrile/H_2_O (90:10, v/v) and filteration through a 0.22 μm membrane filter for liquid chromatography – tandem mass spectrometry (LC−MS/MS) analysis. Gibberellin contents were determined by MetWare (http://www.metware.cn/) on the AB Sciex QTRAP® 6500 + LC−MS/MS platform.

## CONFLICTS OF INTEREST

The authors declare no conflicts of interest.

## AUTHOR CONTRIBUTIONS

H.Q., R.H., and R.Q. conceived the project and analyzed the data. D.X., Y.L., B.G., Z.Z., Z.S., and J.W. performed the experiments. C.Y. and Z.Q. provided useful suggestions. H.Q. and R.H. wrote the manuscript. All authors have read and approved the contents of this paper.

## Supporting information

Additional Supporting Information may be found online in the supporting information tab for this article: http://onlinelibrary.wiley.com/doi/10.1111/jipb.70062/suppinfo

**Figure S1.** OsSAE1 positively regulates seed germination by enhancing α‐amylase activity
**Figure S2.** *OsSLR1* expression is not regulated by OsSAE1
**Figure S3.** OsSAE1 possesses both transcriptional activation and repression activities
**Figure S4.** Identification of the *ks1* mutant and *OsGA2ox3* overexpression lines
**Figure S5.** Knocking out *OsGA2ox3* does not affect seed germination and GA content
**Figure S6.** OsSLR1 represses GA‐induced seed germination
**Figure S7.** OsSLR1 does not affect the binding of OsABI5 to the *OsPRR95* promoter
**Figure S8.** OsSAE1 promotes seed germination in freshly harvested mature panicles
**Figure S9.** Natural variation in the *OsSAE1* coding region is not responsible for variation in seed germination rate
**Table S1.** List of primers used in this study

## Data Availability

Sequence data from this article can be found in the Rice Genome Annotation Project website (http://rice.plantbiology.msu.edu/) under the following accession numbers: *OsSAE1*, LOC_Os06g43220; *OsABI5*, LOC_Os01g64000; *OsSLR1*, LOC_Os03g49990; *OsPRR95*, LOC_Os09g36220; *OsKS1*, LOC_Os04g52230; *OsGA2ox3*, LOC_Os01g55240; *OsCAT2*, LOC_Os02g02400; *OsAPX1*, LOC_Os03g17690.
