# Supplementary material for: OsSAE1 orchestrates the antagonistical regulation of gibberellin and abscisic acid signaling to control rice seed germination
Source: J Integr Plant Biol. 2025 Oct 30;68(1):203–19. doi: 10.1111/jipb.70062 (PMC12782891; doi:10.1111/jipb.70062)
Supplement: Supplementary file 1 — Figure S1. OsSAE1 positively regulates seed germination by enhancing α‐amylase activity Figure S2. OsSLR1 expression is not regulated by OsSAE1 Figure S3. OsSAE1 possesses both transcriptional activation and repression activities Figure S4. Identification of the ks1 mutant and OsGA2ox3 overexpression lines Figure S5. Knocking out OsGA2ox3 does not affect seed germination and GA content Figure S6. OsSLR1 represses GA‐induced seed germination Figure S7. OsSLR1 does not affect the binding of OsABI5 to the OsPRR95 promoter Figure S8. OsSAE1 promotes seed germination in freshly harvested mature panicles Figure S9. Natural variation in the OsSAE1 coding region is not responsible for variation in seed germination rate Table S1. List of primers used in this study [file JIPB-68-203-s001.docx]

**Supporting information**

**OsSAE1 orchestrates the antagonistical regulation of gibberellin and abscisic acid signaling to control rice seed germination**


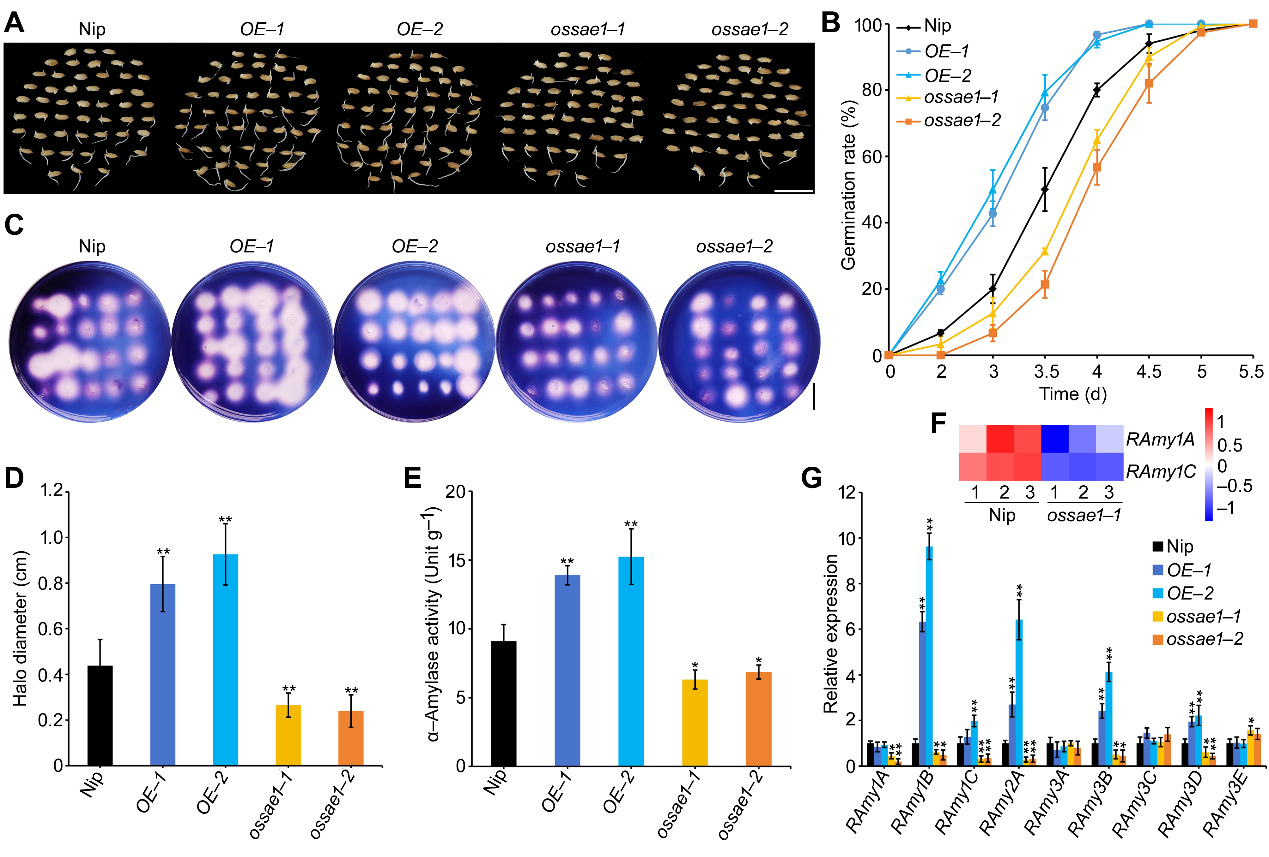


**Figure S1. OsSAE1 positively regulates seed germination by enhancing α-amylase activity.**

**(A)** Representative images of seed germination performance of Nipponbare (Nip), *ossae1* mutants, and *OsSAE1-OE* lines at 3 d. Scale bar, 1 cm. **(B)** Dynamic of germination rate among Nip, *ossae1* mutants, and *OsSAE1-OE* lines during seed germination. The data are shown as mean ± *SD*, *n* = 3 biological replicates. **(C)** Representative images of qualitative comparisons of α-amylase activity between Nip, *ossae1* mutants, and *OsSAE1-OE* seeds at 48 h after incubation in the starch plate test. Scale bar, 1 cm. **(D)** Quantitative analysis of the halo diameters in **(C)**. The data are shown as mean ± *SD*, *n* = 20 halos. **(E)** Quantitative analysis of α-amylase enzyme activity in Nip, *ossae1* mutants, and *OsSAE1-OE* seeds at 48 h after imbibition. The data are shown as mean ± *SD*, *n* = 3 biological replicates. **(F)** Heat map of microarray expression proﬁles for *RAmy* genes. **(G)** Expression of α-amylase family genes in the germinating (2 d after imbibition) seeds in Nip, *ossae1* mutants, and *OsSAE1-OE* lines. *Actin1* was used as an internal control. The relative expression levels were represented by fold change relative to the expression levels of Nip. Data were presented as mean ± *SD*, *n* = 3 biological replicates. For **(D)**, **(E)**, and **(G)**, asterisks indicate signiﬁcant differences compared with Nip at **P* < 0.05 and ***P* < 0.01 (Student’s *t* test).


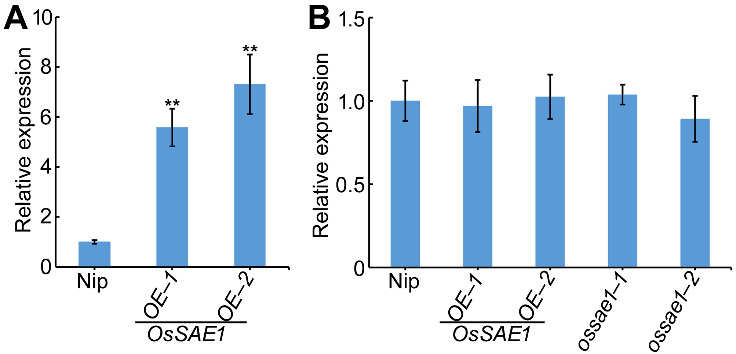


**Figure S2. The expression of *OsSLR1* was not regulated by OsSAE1.**

**(A)** Expression of *OsSAE1* in the germinating (2 d after imbibition) seeds in Nip and *OsSAE1-OE* lines. **(B)** Expression of *OsSLR1* in the germinating (2 d after imbibition) seeds in Nip, *ossae1* mutants, and *OsSAE1-OE* lines. For **(A)** and **(B)**, *Actin1* was used as an internal control. The relative expression levels were represented by fold change relative to the expression levels of Nip. Data were presented as mean ± *SD*, *n* = 3 biological replicates. Asterisks indicate signiﬁcant differences compared with Nip at ***P* < 0.01 (Student’s *t* test).


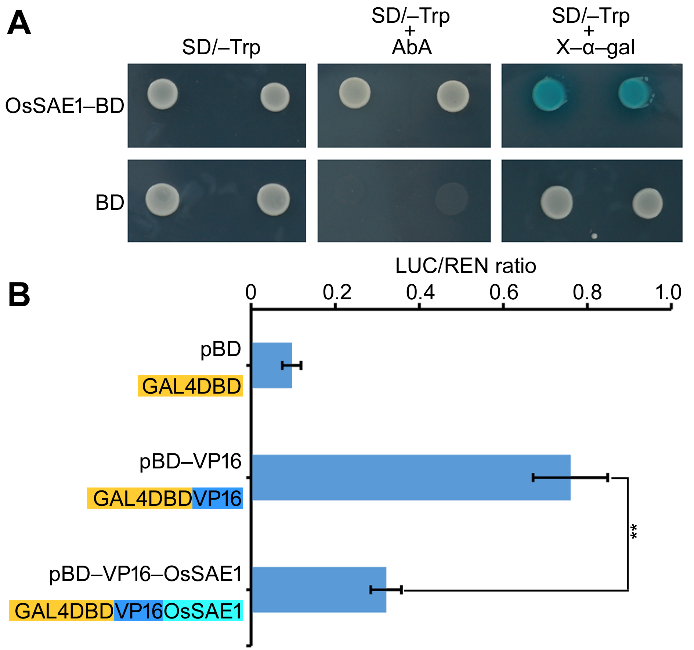


**Figure S3. OsSAE1 possesses both transcriptional activation and repression activities simultaneously.**

**(A)** Transcriptional activity analysis of OsSAE1 in yeast cells. Transformed yeast cells were spotted on the control medium (SD/−Trp) and selective medium (SD/−Trp + AbA or SD/−Trp + X-α-gal). **(B)** Transient dual-LUC expression assays illustrating the transcriptional repression activity of OsSAE1. The reporters LUC and REN and the effectors (pBD, pBD-VP16, and pBD-VP16-OsSAE1) were separately constructed as shown on the left. The activities of LUC and REN were determined 16 h after transformation, and the relative LUC/REN ratios represent the transcriptional activation activities. Data were presented as mean ± *SD*, *n* = 3 biological replicates. Asterisks indicate signiﬁcant differences between the compared two samples at ***P* < 0.01 (Student’s *t* test).


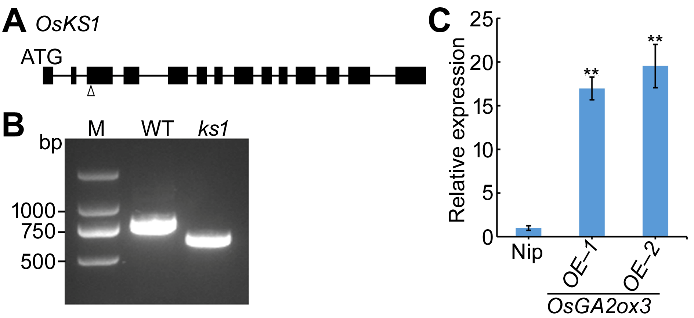


**Figure S4. Identification of *ks1* mutant and** ***OsGA2ox3* overexpression lines.**

**(A)** Mutation sites of *ks1* mutant shown on the schematic diagram of *OsKS1*. **(B)** Confirmation of mutation sites in *ks1* by PCR-based analyses. **(C)** Expression of *OsGA2ox3* in the germinating (2 d after imbibition) seeds in ZH11 and *OsGA2ox3-OE* lines. *Actin1* was used as an internal control. The relative expression levels were represented by fold change relative to the expression levels of ZH11. Data were presented as mean ± *SD*, *n* = 3 biological replicates. Asterisks indicate signiﬁcant differences compared with ZH11 at ***P* < 0.01 (Student’s *t* test).


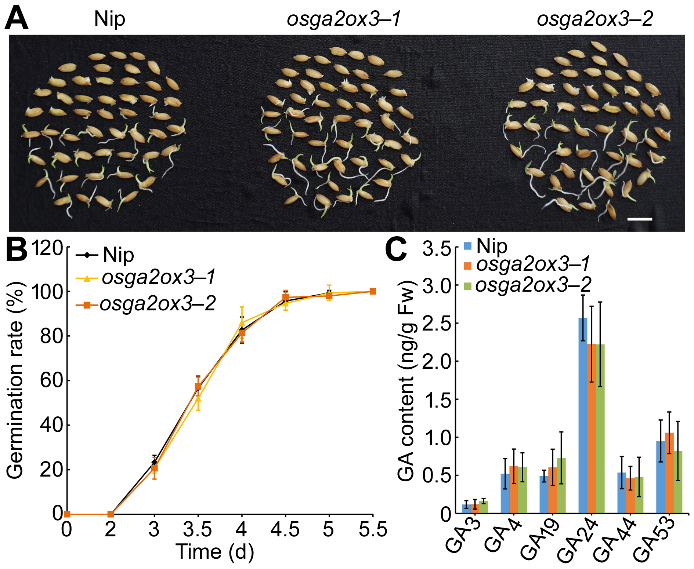


**Figure S5. Knocking out *OsGA2ox3* does not affect seed germination and GA contents.**

**(A)** Representative images of seed germination performance of Nip and *osga2ox3* mutants at 3.5 d. Scale bar, 1 cm. **(B)** Dynamic of germination rate among Nip and *osga2ox3* mutants during seed germination. The data are shown as mean ± *SD*, *n* = 3 biological replicates. **(C)** Content of various GAs in the germinating (2 d after imbibition) seeds in Nip and *osga2ox3* mutants. Data were presented as mean ± *SD*, *n* = 3 biological replicates. Fw, fresh weight.


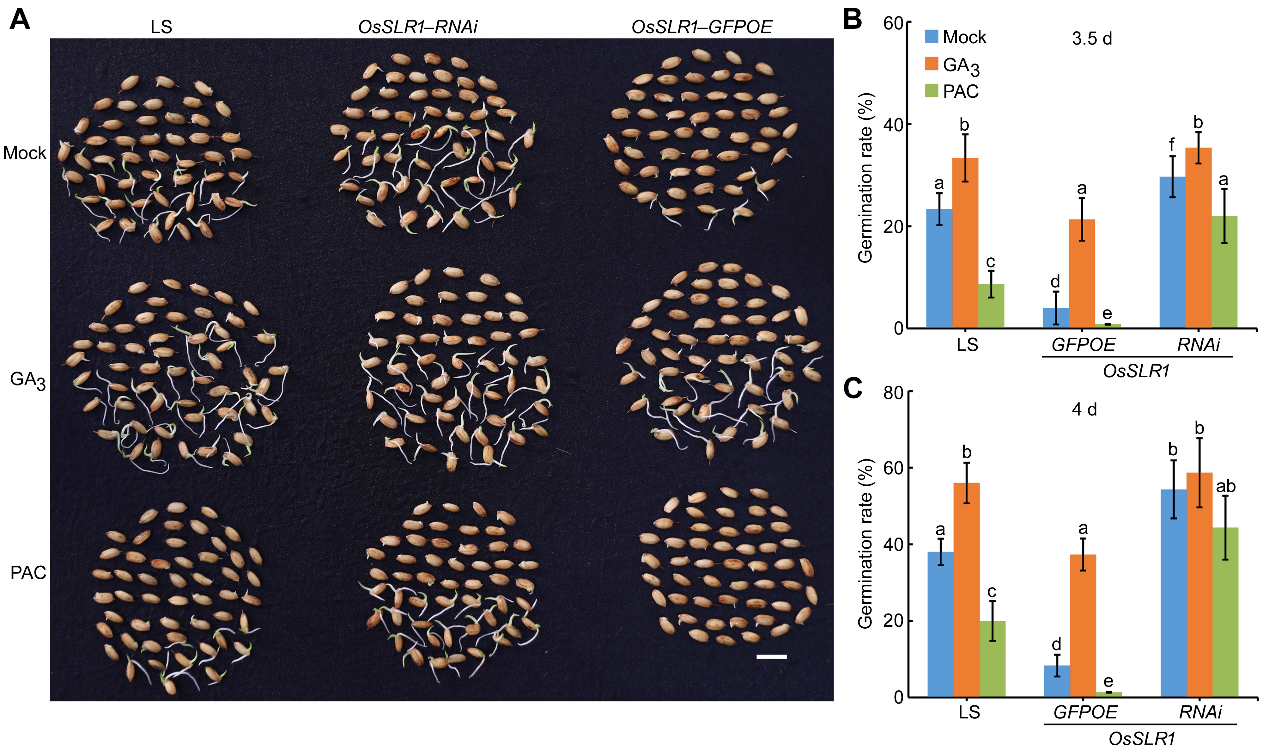


**Figure S6. OsSLR1 mediates GA-promoted seed germination.**

**(A)** Representative images of seed germination performance of Lansheng (LS), *OsSLR1-RNAi* and *OsSLR1-GFPOE* lines with or without 5 μM GA_3_ or 2 μM PAC treatment for 3.5 d. Scale bar, 1 cm. **(B**, **C)** Germination rate of seeds in **(A)** at 3.5 d **(B)** and 4 d **(C)**. The data are shown as mean ± *SD*, *n* = 3 biological replicates. Different letters indicate signiﬁcant differences (*P* < 0.05, one-way ANOVA with Tukey’s test).


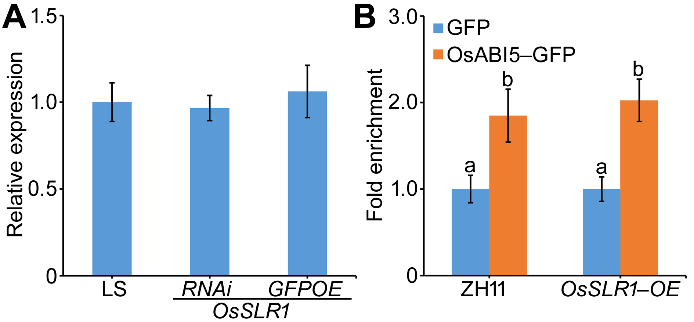


**Figure S7. OsSLR1 does not affect the binding of OsABI5 to *OsPRR95*.**

**(A)** Expression of *OsABI5* in the germinating (2 d after imbibition) seeds in LS, *OsSLR1-RNAi* and *OsSLR1-GFPOE* lines. *Actin1* was used as an internal control. The relative expression levels were represented by fold change relative to the expression levels of LS. Data were presented as mean ± *SD*, *n* = 3 biological replicates. **(B)** tsCUT&Tag-qPCR analysis of OsABI5 binding to *OsPRR95* in ZH11 and *OsSLR1-OE* lines. The data are shown as mean ± *SD*, *n* = 3 biological replicates. Different letters indicate signiﬁcant differences (*P* < 0.05, one-way ANOVA with Tukey’s test).


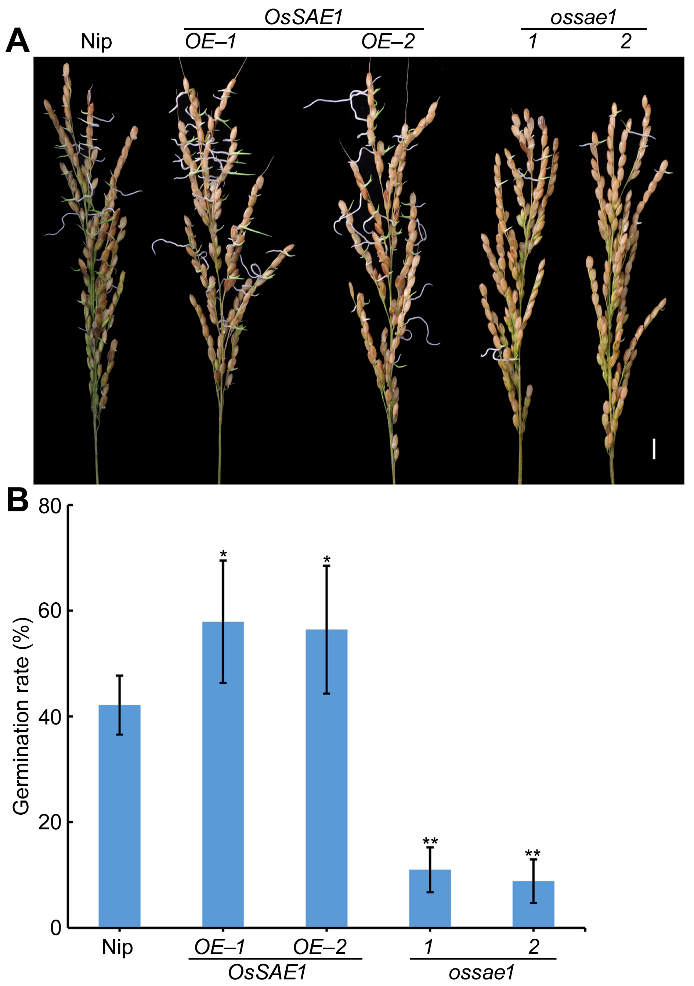


**Figure S8. OsSAE1 positively regulates seed germination in freshly harvested mature panicles.**

**(A)** Germination performance of seeds in freshly harvested mature panicles of Nip, *ossae1* mutants, and *OsSAE1-OE* lines. Scale bar, 1 cm. **(B)** Germination percentage of seeds in freshly harvested mature panicles of Nip, *ossae1* mutants, and *OsSAE1-OE* lines at 7 d after imbibition. The data are shown as mean ± *SD*, *n* = 3 biological replicates. Asterisks indicate signiﬁcant differences compared with Nip at **P* < 0.05 and ***P* < 0.01 (Student’s *t* test).


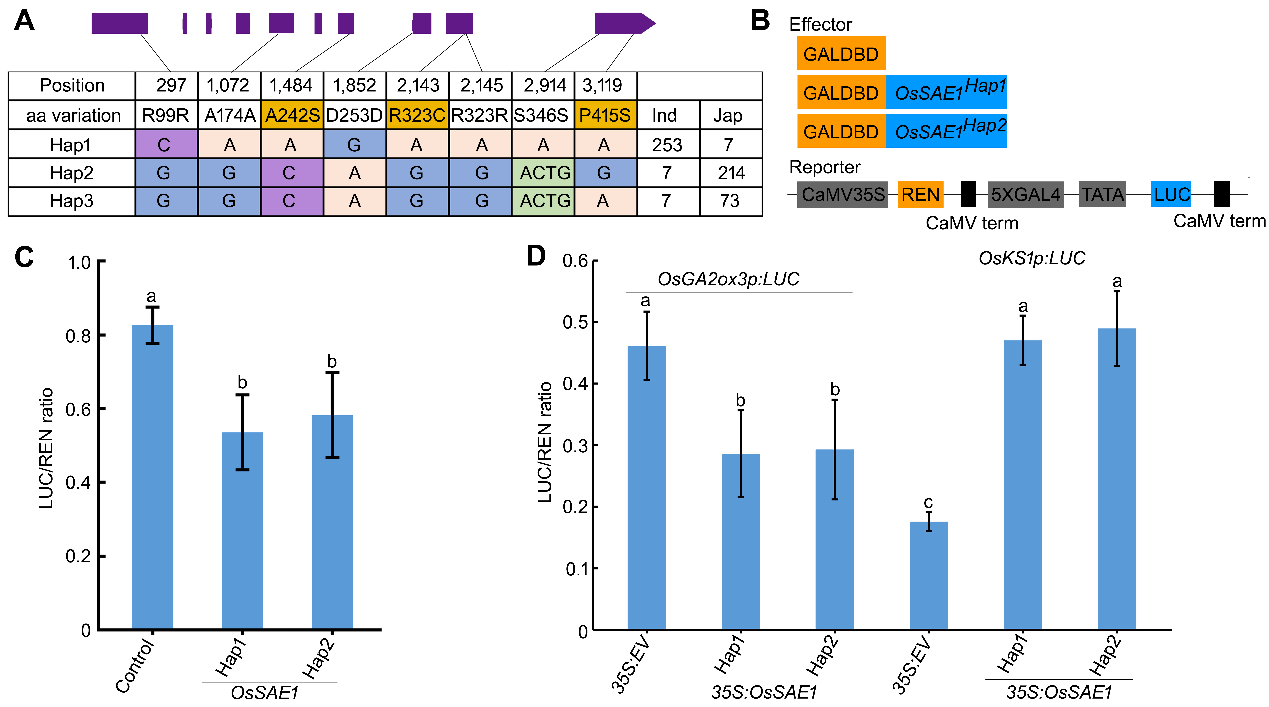


**Figure S9. Natural variation in *OsSAE1* coding region is not responsible for the variation of seed germination.**

**(A)** Haplotype analysis of *OsSAE1* coding regions from 578 rice cultivars. **(B)** Schematic diagrams of reporter and effector constructs used in the protoplast transcription system. **(C)** Transcriptional activity assays of the haplotypes (*OsSAE1^Hap1^* and *OsSAE1^Hap2^*) of *OsSAE1* coding regions. **(D)** Transcriptional activity assays of the haplotypes (*OsSAE1^Hap1^* and *OsSAE1^Hap2^*) of *OsSAE1* coding regions on downstream genes.For **(C)** and **(D)**, Data were presented as mean ± *SD*, *n* = 3 biological replicates. Different letters indicate signiﬁcant differences (*P* < 0.05, one-way ANOVA with Tukey’s test).

**Table S1. Primers used in this study.**

| **Primer** | **Sequence** (5'-3') |
| --- | --- |
| **qPCR analysis** | |
| *OsActin1*-F | GACCTTGCTGGGCGTGAT |
| *OsActin1*-R | GTCATAGTCCAGGGCGATGT |
| *OsSAE1-F* | CTTGGCCTGTATGACACGGA |
| *OsSAE1-R* | AGATTGCAGGTTGAGCTCGT |
| *OsABI5-F* | CTCGTCTCAAAGAGGCAGAGAAG |
| *OsABI5-R* | TCTTCTCCTTGGACTGCTCCATC |
| *OsKAO-F* | TTCGCAAGAGCAAAGGCTGAG |
| *OsKAO-R* | GGAAGGACACGAAGGAGATG |
| *OsCPS1-F* | ATGTCAAGCATGAAAAGGATGG |
| *OsCPS1-R* | ACTTTTGACCTTCTCGAACTG |
| *OsKO2-F* | TAGCCAAGGAGGCGATGGTTG |
| *OsKO2-R* | TGTCACGAAATTGTTTCTGTGC |
| *OsKS1-F* | TATGTGCCGACAATGGAAGA |
| *OsKS1-R* | CTGTTCAGCTTTCCCTCCAG |
| *OsGA20ox2-F* | CAATGGGGAGGGTGTACCAGA |
| *OsGA20ox2-R* | TCCTGGAGGAGGATGGTGAG |
| *OsGA2ox3-F* | AGCACAGAGGATTGCACCAT |
| *OsGA2ox3-R* | CAGCCTGTTGTCTCCAAGCC |
| *EUI1-F* | CAAGTGGAGGAAAGCCTACG |
| *EUI1-R* | GACACGGGCCATGTAGAACT |
| *GID1-F* | CTCCAGGACAGGGACTGGTA |
| *GID1-R* | CATTGGACAACCTTGACGTG |
| *OsKOS1-F* | CCCAAGGACGAAATCTTTGA |
| *OsKOS1-R* | TGCAATCCTCTCCTTCTGCT |
| *RAmy1A-qF* | GGTCAACTGGGTCGATCGT |
| *RAmy1A-qR* | CTGCCGGTTTCTGATTGAC |
| *RAmy1B-qF* | TGGTGGCGAACAAACACTTC |
| *RAmy1B-qR* | CACCTAGCTTGCCCATAAGC |
| *RAmy1C-qF* | GCTAAGGCGACGACCTTCA |
| *RAmy1C-qR* | CGTCGTATCTTGGTCCGAC |
| *RAmy2A-qF* | GCGGCTGATCGACCAGC |
| *RAmy2A-qR* | GTCGTGCAGCATGATCTTG |
| *RAmy3A-qF* | TCGTGAACTGGGTGAAGCA |
| *RAmy3A-qR* | CCCGTTGCGCTTCCTCGT |
| *RAmy3B-qF* | TCGGTGGCCCTGCGTCA |
| *RAmy3B-qR* | CATTTCTTGATCTCACTGCAG |
| *RAmy3C-qF* | TCGGTGGCCCTGCATCG |
| *RAmy3C-qR* | TTCTTGACCTCACCGCGG |
| RAmy3D-qF | GGCTCGACGCAGAAGCTT |
| RAmy3D-qR | GACGACGGCGACGTATGC |
| *RAmy3E-qF* | GCTCGACCCAGTCGCTCT |
| *RAmy3E-qR* | CACGACATGGAAGTCGGAG |
| *OsPRR95-F* | ACGAGAGGCTGCACTGAACA |
| *OsPRR95-R* | TGGACACCATGATCTTGCCGAA |
| *OsAPX1-F* | GACATTGTTGCCCTCTCTGG |
| *OsAPX1-R* | GGGCTTTGTCACTAGGAAGC |
| *OsCAT2-F* | CCTATGCTGATACCCAAAG |
| *OsCAT2-R* | CACACTGCGACCAGTAGGA |
| *OsSLR1-F* | CAGGTCATGTCCGAGGTGTA |
| *OsSLR1-R* | TTGTAGGCATTGGAGCCCAG |
| **For *ks1* mutant identification** | |
| KS1-JD-F | CTTGGTGCCTCGTGTCTT |
| KS1-JD-R | CAAGGCTAAGCATACCAG |
| KS1-JD-T | CGGTTACATCTTCTCAAACTCAATGTG |
| **For Pull-down** | |
| OsSLR1-30a-F | GCCATGGCTGATATCGGATCCATGAAGCGCGAGTACCAAG |
| OsSLR1-30a-R | GCAAGCTTGTCGACGGAGCTCCGCCGCGGCGACGCGCCAT |
| OsABI5-GST-F | CCCCTGGGATCCCCGGAATTCATGGCATCGGAGATGAGCA |
| OsABI5-GST-R | GTCACGATGCGGCCGCTCGAGCCACATGCAGCTGCCGCTG |
| **For BiFc** | |
| OsABI5-nYFP-F | GAACACGGGGGGACTCTAGAATGGCATCGGAGATGAGCAA |
| OsABI5-nYFP-R | AACTTTTGCTCCATCCCGGGCCACATGCAGCTGCCGCTG |
| OsSLR1-cYFP-F | AGAACACGGGGGACTCTAGAATGAAGCGCGAGTACCAAGA |
| OsSLR1-cYFP-R | TCGTATGGGTACATCCCGGGCGCCGCGGCGACGCGCCATG |
| **For Y1H** | |
| OsSAE1-AD-F | CCATGGAGGCCAGTGAATTCATGGCGGCAACCTTCTACG |
| OsSAE1-AD-R | AGCTCGAGCTCGATGGATCCGCGGCCAGTAGTTGCAG |
| pAbAi-pOsKS1-F | TTGAATTCGAGCTCGGTACCTATCATCGAGTTGGGCCGCA |
| pAbAi-pOsKS1-R | GCACATGCCTCGAGGTCGACCGGCAGGAGGAGGAGGAGGA |
| pAbAi-pOsGA2OX3-F | TTGAATTCGAGCTCGGTACCGTCCACCGTTCCCTCAGCAA |
| pAbAi-pOsGA2OX3-R | GCACATGCCTCGAGGTCGACGGCCAATAGCGTGTGCGAGG |
| **For Y2H** | |
| OsSLR1-BD-F | TGGCCATGGAGGCCGAATTCATGAAGCGCGAGTACCAAG |
| OsSLR1-BD-R | CGCTGCAGGTCGACGGATCCCGCCGCGGCGACGCGCCAT |
| OsSAE1-BD-F | TGGCCATGGAGGCCGAATTCATGGCGGCAACCTTCTACG |
| OsSAE1-BD-R | CGCTGCAGGTCGACGGATCCGCGGCCAGTAGTTGCAG |
| OsABI5-AD-F | GCCATGGAGGCCAGTGAATTCATGGCATCGGAGATGAGCA |
| OsABI5-AD-R | CCTAGGTAGCTCGAGCTCGACCCACATGCAGCTGCCGCTG |
| **For dual-luciferase assays** | |
| 35S-OsSAE1-F | GTTTGGTGTTACTTCTGCAGATGGCGGCAACCTTCTACGGCGT |
| 35S-OsSAE1-R | TCACACGTGAGCTCGGATCCGCGGCCAGTAGTTGCAGTGGCAG |
| 35S-OsABI5-F | GTTTGGTGTTACTTCTGCAGATGGCATCGGAGATGAGCAAGA |
| 35S-OsABI5-R | TCACACGTGAGCTCGGATCCCCACATGCAGCTGCCGCTGCGG |
| 35S-OsSLR1-F | GGACAGCCCAGATCAACTAGTATGAAGCGCGAGTACCAAGAAGCC |
| 35S-OsSLR1-R | GCTCACCATGGATCCCCCGGGCGCCGCGGCGACGCGCCATG |
| OsSAE1-VP16-F | ATGGATCCTTATGGCGGCAACCTTCTACGGCGT |
| OsSAE1-VP16-R | ATGGTACCGCGGCCAGTAGTTGCAGTGGCAG |
| *OsKS1p*-LUC-F | TAAGCTTGATATCGAATTCCTGCAGGCATGTAGGGTATCATCGA |
| *OsKS1p*-LUC-R | CGGCCGCTCTAGAACTAGTGGATCCAGGAGGAGGAGATGACACGGCACAA |
| *OsGA2ox3p*-LUC-F | TAAGCTTGATATCGAATTCCTGCAGGTCGACCTGTACCACTGGAATCTCGAA |
| *OsGA2ox3p*-LUC-R | CGGCCGCTCTAGAACTAGTGGATCCACTAGTGGCCAATAGCGTGTGCGAGG |
| *OsPRR95p*-LUC-F | TAAGCTTGATATCGAATTCCTGCAGGGACCATGTATTAATAGGGCAGTAT |
| *OsPRR95p*-LUC-R | CGGCCGCTCTAGAACTAGTGGATCCCCTCCCCTTCCTCCCCGTCCCCGTC |
| *OsSAE1p*-LUC-F | TAAGCTTGATATCGAATTCCTGCAGTACACCAACTACTCATTTCCTA |
| *OsSAE1p*-LUC-R | CGGCCGCTCTAGAACTAGTGGATCCGGCCTACTACACACCGGCGTTCT |
| **For tsCUT&Tag-qPCR** | |
| OsSAE1-GFP-F | AAGTCCGGAGCTAGCTCTAGAATGGCGGCAACCTTCTACG |
| OsSAE1-GFP-R | GCCCTTGCTCACCATGGATCCGCGGCCAGTAGTTGCAGTG |
| OsABI5-GFP-F | AAGTCCGGAGCTAGCTCTAGAATGGCATCGGAGATGAGCA |
| OsABI5-GFP-F | GCCCTTGCTCACCATGGATCCCCACATGCAGCTGCCGCTG |
| OsKS1-P1F | TCAACTTCTTTAGTAGCGGG |
| OsKS1-P1R | CGACGGTCTTTATCACCA |
| OsKS1-P2F | TGTTCCATCCACATCGCC |
| OsKS1-P2R | CTTCCGCTTGCTATTGGC |
| OsGA2ox3-P1F | TAGTTGCTTCAGCGGCAT |
| OsGA2ox3-P1R | TGAGGGAACGGTGGACTT |
| OsGA2ox3-P2F | CTTCCATAGGTTTCACCC |
| OsGA2ox3-P2R | CTTGCTTAGTTAGGTCACCA |
| OsGA2ox3-P3F | GTTGTATTACTTACCTCCTTGC |
| OsGA2ox3-P3R | TCATAGTCTTCACGCCAG |
| OsPRR95-P1F | ATATTTCGTGCGCGGAGCTC |
| OsPRR95-P1R | GGGGGAGGAGGCGTGTGTATATA |
| **For EMSA** | |
| OsSAE1-GST-F | GGATCCATGGCGGCAACCTTCTACGGCGT |
| OsSAE1-GST-R | CTCGAGGCGGCCAGTAGTTGCAGTGGCAG |
| OsKS1-Probe 1 | CTGATTTTTGCCCGAGGCACGGCCGCCACAGCCTCTAGGAGTGCACCTG |
| OsKS1-Probe 2 | CTCGCCGGCGCGCCGCCCCTCTCCGGCCCTGCGCCGCCGTGGTCGCCCTA |
| OsKS1-Probe 3 | CTCCGGCATCCACTGTCGGCGGGCGGCCGCGAAAAAGGTGCGTTTCCTA |
| OsGA2ox3-Probe 1 | AACTGGCCTGCCGGCAGGCAGGCCGCCACCGTTGATCGCCGCGAAAAAG |
| OsGA2ox3-Probe 2 | AGAACCGGAGAAGAGAGCAATGGCGGCAATGCTTAATTATCTGCTGGAG |
| OsGA2ox3-Probe 3 | CAGGGGCGGATCCAGCATGGGGGCGGCGGGGTCTCGAGCCCCCACTACT |
